# Anti-myeloma mechanisms of the selective NF-κB inhibitor QNZ

**DOI:** 10.1016/j.omton.2026.201267

**Published:** 2026-06-12

**Authors:** Dana Cholujova, Zuzana Valuskova, Monika Burikova, Milan Hucko, Gabor Beke, Eva Sedlackova, Lubos Klucar, Katarina Suroviakova, Gabriela Grofova, Jan Sedlak, Jana Jakubikova

**Affiliations:** 1 Cancer Research Institute, Biomedical Research Center, v. v. i., Slovak Academy of Sciences, Dubravska cesta 9, 84505 Bratislava, Slovakia; 2 Institute of Molecular Biology, Slovak Academy of Sciences, Dubravska cesta 21, 84551 Bratislava, Slovakia; 3 Centre for Advanced Materials Application, Slovak Academy of Sciences, Dubravska cesta 9, 84511 Bratislava, Slovakia

**Keywords:** multiple myeloma, the selective NF-κB inhibitor QNZ, EVP4593, NF-κB signaling, tumor microenvironment

## Abstract

Multiple myeloma (MM) is a plasma-cell malignancy driven by dysregulated NF-κB signaling, promoting tumor survival, proliferation, and chemoresistance. Given observed overexpression of NF-κB components in MM cell lines, we evaluated the selective NF-κB inhibitor QNZ (EVP4593) for anti-myeloma activity. QNZ selectively reduced viability and inhibited proliferation of MM cells *in vitro* and *ex vivo*, though stromal contact partly attenuated its cytotoxicity. QNZ dose-dependently suppressed MM xenografts, increased apoptosis, and reduced proliferation, while preserving MM plasma cell identity. Mechanistically, QNZ triggered mitochondrial, caspase- (cleavage of pro-caspase-9, -8 and -3) and PARP-mediated apoptosis with downregulation of Mcl-1, dismantled pro-survival NF-κB/Akt/c-Myc signaling with concomitant mTOR modulation, and induced cell-cycle perturbation accompanied by altered phospho-ATM and decreased levels of key regulators (SIRT1, Chk2/p-Chk2, Cdc2/p-Cdc2, p-4EBP1, CDK4, cyclin A2, and cyclin B1). Transcriptomic profiling demonstrated that QNZ treatment induced extensive gene expression reprogramming in MM cells, prominently upregulating stress- and metabolism-associated genes including DDIT3, PHGDH, SESN2, and NFE2L1, consistent with activation of ER stress-mediated apoptotic and amino acid metabolic pathways. Finally, QNZ displayed significant synergy with proteasome inhibitors and IMiDs, particularly second-generation carfilzomib and pomalidomide, as well as with dexamethasone and melphalan, providing a strong preclinical rationale for clinical evaluation of QNZ in MM.

## Graphical abstract

**Figure:**
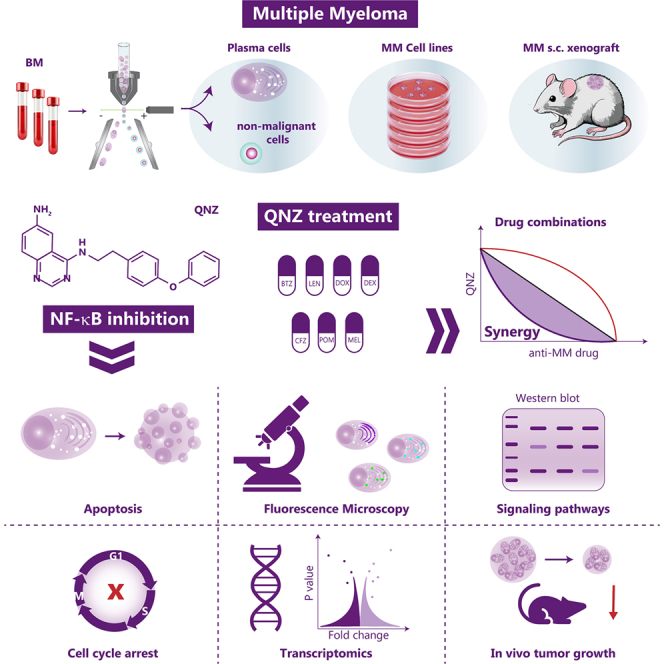

## Teaser

QNZ (EVP4593), a selective NF-κB inhibitor, selectively kills myeloma cells while sparing healthy counterparts, suppresses tumor growth *in vivo*, and synergizes with approved myeloma therapies. These findings establish NF-κB inhibition as a promising and combinable therapeutic strategy in multiple myeloma.

## Introduction

Nuclear factor kappa B (NF-κB) signaling is a pivotal driver of multiple myeloma (MM) pathogenesis, disease progression, and therapeutic resistance. MM is hallmarked by the clonal expansion of malignant plasma cells (PCs) within the bone marrow, where both the canonical and non-canonical NF-κB pathways sustain tumor cell survival, proliferation, drug resistance, and crosstalk with the microenvironment.^1^^,^^2^ The canonical NF-κB pathway is activated by pro-inflammatory cytokines (e.g., TNF-α, IL-1β), growth factors, and receptor ligation (TACI, BCMA), culminating in nuclear translocation of p65/RelA-p50 dimers. In contrast, the non-canonical pathway is engaged by BAFF and CD40L through NIK stabilization, promoting p52/RelB activation.^1^^,^^3^ Recurrent mutations in NF-κB regulators such as TRAF3, CYLD, MAP3K14 (NIK), NFKB1, and NFKB2 occur in approximately 17% of primary MM tumors and 40% of cell lines, correlate with advanced disease stage and poor prognosis, and drive activation of the canonical and/or non-canonical NF-κB pathways, thereby promoting disease progression and drug resistance.^4^ Beyond genetic drivers, the MM bone marrow niche, especially inflammatory mesenchymal stromal cells (iMSCs), secretes cytokines such as BAFF and APRIL to sustain and amplify NF-κB signaling. Enriched in patient marrow, iMSCs exhibit NF-κB-dependent transcriptional programs that drive tumor growth and chemoresistance, although only a subset of MM cells exhibits high pathway activation in response to these microenvironmental cues.^1^^,^^5^ These insights underscore the therapeutic potential of targeting NF-κB signaling with pathway-specific inhibitors to overcome resistance and improve outcomes in MM.

Current clinical treatment regimens for MM rely on proteasome inhibitors, immunomodulatory drugs (IMiDs), corticosteroids, and monoclonal antibodies, many of which exert indirect inhibitory effects on NF-κB signaling. Proteasome inhibitors such as bortezomib (BTZ) prevent the degradation of IκB, thereby blocking NF-κB nuclear translocation, while immunomodulatory agents like thalidomide and lenalidomide (LEN) suppress pro-inflammatory cytokines including TNFα, IL1β, and IL6 that drive NF-κB activation.^6^^,^^7^ Specific quinazoline-derived NF-κB inhibitor QNZ (EVP4593) blocks NF-κB activation, downregulates metastasis-associated proteins and induces apoptosis in tumor cells.^8^ It synergizes with BMP2 to modulate fibroblast proliferation and migration, thereby promoting osteogenesis and potentially remodeling the tumor microenvironment.^9^ Extending its NF-κB–inhibitory core across diverse cancers, QNZ abrogates MEX3A-driven NF-κB signaling in nasopharyngeal carcinoma,^10^ reducing proliferation and invasion, and suppresses EAAT3 expression in lung cancer cells to impair antioxidant defenses and reprogram tumor metabolism.^11^ In CD146-overexpressing hepatocellular carcinoma cells, QNZ-mediated NF-κB blockade downregulates JAG2, NOTCH1, and HES1, attenuating stemness and chemoresistance.^12^ High-throughput screening has identified QNZ as a sensitizer to glucose starvation and VEGF inhibition *in vitro* and *in vivo*,^13^ and in a rat hepatocellular carcinoma model, it reduces tumor growth by targeting inflammatory pathways.^14^ Moreover, a related quinazoline analog induces reactive oxygen species (ROS)-dependent G_2_/M arrest and apoptosis in lung cancer cells, underscoring the scaffold’s versatility.^15^ These collective findings justify the preclinical evaluation of QNZ as a targeted NF-κB inhibitor in MM to overcome both intrinsic genetic lesions and microenvironment-driven signaling that underlie disease progression and therapeutic resistance.

In our study, we evaluated the selective NF-κB inhibitor QNZ (EVP4593) across cellular, *ex vivo*, and xenograft models of MM to define its anti-myeloma activity and underlying mechanisms. We demonstrate that QNZ selectively suppresses NF-κB-driven survival signaling, reduces viability of MM cell lines, and patient-derived PCs while largely sparing healthy counterparts, and retains activity in bone marrow stromal co-cultures despite partial attenuation. QNZ inhibited myeloma tumor growth *in vivo* in a dose-dependent manner, with maximal antitumor efficacy observed at the highest dose tested. Mechanistically, QNZ induces mitochondrial, caspase-dependent apoptosis, downregulates Akt and c-Myc, perturbs key cell-cycle regulators, SIRT1, Chk2/p-Chk2, Cdc2/p-Cdc2, p-4EBP1, CDK4, cyclin A2 and cyclin B1, and alters phospho-ATM, consistent with coordinated disruption of pro-survival and proliferative pathways. Transcriptomic profiling revealed that QNZ treatment induced broad gene expression reprogramming in MM cells, characterized by the coordinated regulation of stress response, amino acid metabolism, and endoplasmic reticulum (ER) stress-related apoptotic pathways. Finally, QNZ synergizes with proteasome inhibitors and IMiDs (particularly the second-generation agents, carfilzomib [CFZ] and pomalidomide [POM]), as well as with conventional agents such as dexamethasone (DEX) and melphalan (MEL), providing a strong preclinical rationale for advancing QNZ in clinical combination studies for MM.

## Results

### QNZ exhibits time- and dose-dependent *in vitro* cytotoxicity in MM models

**Figure 1:**
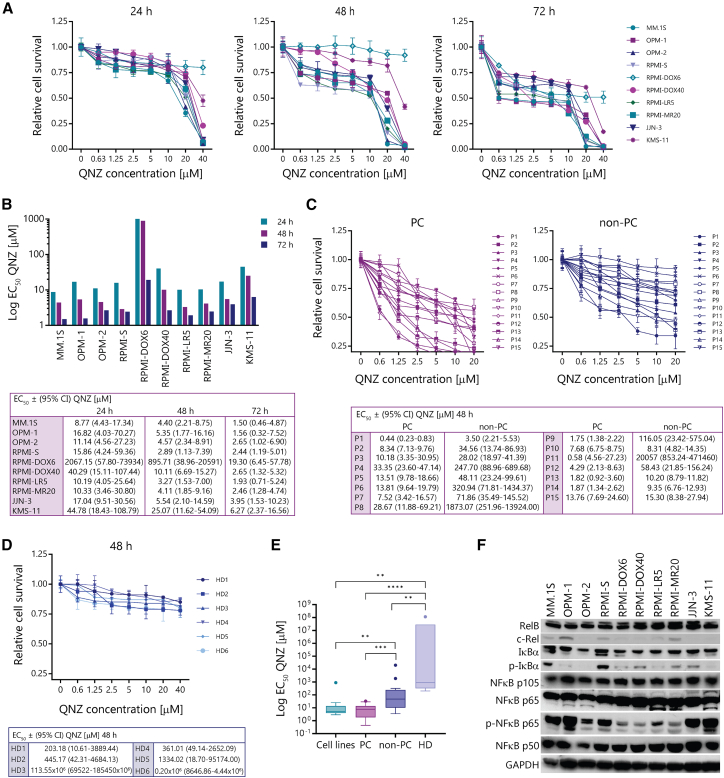
QNZ cytotoxicity in MM cell lines and primary cells (A) Dose-response curves for QNZ (0.625–40 μM; 24, 48, and 72 h) in MM cell lines (MM.1S, OPM-1, OPM-2, RPMI-S, RPMI-DOX6, RPMI-DOX40, RPMI-LR5, RPMI-MR20, JJN-3, and KMS-11). (B) Half-maximal effective concentrations (EC_50_) with 95% confidence intervals (CI) for QNZ in each MM line at 24, 48, and 72 h. (C) EC_50_ values (with 95% CI) for QNZ (0.625–20 μM; 48 h) in freshly sorted malignant plasma cells (PC; CD138^+^/CD45low/CD38^++^; *n* = 15) and non-plasma mononuclear cells (non-PC; *n* = 15) from MM patients. (D) EC_50_ values (with 95% CI) for QNZ (0.625–40 μM; 48 h) in peripheral blood mononuclear cells from healthy donors (HD; *n* = 6). Data are presented as mean ± SD relative to untreated controls. (E) Comparison of median log EC_50_ values for QNZ across MM cell lines, PC, non-PC, and HD. Statistical significance: ∗*p* < 0.05; ∗∗*p* < 0.01; ∗∗∗*p* < 0.001; ∗∗∗∗*p* < 0.0001 (Kruskal-Wallis multiple comparison test). (F) Baseline expression of NF-κB signaling components (RelB, c-Rel, IκBα, phosphorylated IκBα (*p*-IκBα), NF-κB p105, NF-κB p65, phosphorylated NF-κB p65 (p-p65), and NF-κB p50) by western blot in unstimulated MM cell lines. GAPDH was used as a loading control.

To evaluate the *in vitro* anti-myeloma activity of QNZ, we treated ten MM cell lines (MM.1S, OPM-1, OPM-2, RPMI-S, RPMI-DOX6, RPMI-DOX40, RPMI-LR5, RPMI-MR20, JJN-3, and KMS-11) with QNZ at concentrations ranging from 0 to 40 μM for 24, 48, and 72 h and measured viability by 3-[4,5-dimethylthiazol-2-yl]-2,5-diphenyltetrazolium bromide (MTT) assay. QNZ induced a concentration- and time-dependent decrease in cell survival (Figure 1A), with maximal cytotoxicity achieved at 72 h. Among the cell lines, RPMI-DOX6, RPMI-DOX40, and KMS-11 demonstrated the greatest resistance. Half-maximal effective concentrations (EC_50_) calculated via CalcuSyn software ranged from 8.8 to 44.8 μM at 24 h, 2.9 to 25.1 μM at 48 h, and 1.5 to 6.3 μM at 72 h (excluding the RPMI-DOX6 outlier), reflecting an approximate 3-fold and 7-fold reduction in EC_50_ between 24 and 48 h and between 24 and 72 h, respectively (Figure 1B).

We then assessed the effects of QNZ on primary bone marrow-derived malignant PCs and non-PC mononuclear cells (MNCs) from MM patients (*n* = 15) after 48 h of treatment (Figure 1C). The clinical characteristics of primary MM patient samples used in this study are detailed in Table S1. Malignant PCs exhibited EC_50_ values ranging from 0.4 to 33.4 μM, whereas non-PC MNCs were more resistant, with ten patient samples not reaching 50% inhibition at the maximum tested dose (20 μM). Finally, peripheral blood MNCs from healthy donors (HDs; *n* = 6) remained largely unaffected by QNZ, with no sample achieving 50% growth inhibition at concentrations up to 40 μM (Figure 1D). Statistical comparison revealed that primary patient-derived PC, established MM cell lines, and primary non-PC were significantly more sensitive to QNZ than healthy peripheral blood MNCs (Figure 1E). Likewise, primary PC and MM cell lines exhibited significantly greater sensitivity to QNZ compared to non-PC from the MM tumor microenvironment (Figure 1E).

To elucidate the involvement of NF-κB signaling in MM, we quantified the expression of key NF-κB family subunits and their regulators in a panel of ten MM cell lines by western blot analyses (Figure 1F). Specifically, we assessed total RelB, c-Rel, NF-κB p105, NF-κB p50, and NF-κB p65, including its phosphorylated form (p- NF-κB p65), and concurrently analyzed IκBα and its phosphorylated form (*p*-IκBα). Overall, total protein levels of RelB, IκBα, NF-κB p105, NF-κB p65, and NF-κB p50 were comparable across all MM cell lines. In contrast, higher activation of NF-κB signaling, evidenced by elevated p-NF-κB p65, was observed in MM.1S, OPM-1, RPMI-S, JJN3, and KMS11 cells. Phosphorylation of IκBα was detected in MM.1S and RPMI-S cells (with weaker *p*-IκBα signals in the doxorubicin (DOX)-resistant derivatives RPMI-DOX6, RPMI-DOX40, RPMI-LR5, and RPMI-MR20), as well as in JJN3 cells. Expression of c-Rel was restricted to OPM-1 cells and was present at low levels in MM.1S, RPMI-S, and the resistant RPMI-MR20 line.

Notably, canonical NF-κB components are uniformly expressed in MM, whereas differential phosphorylation of NF-κB p65 and IκBα reveals heterogeneous pathway activation. Collectively, these data demonstrate that QNZ selectively and potently inhibits MM cell survival in a time- and dose-dependent manner, with malignant PCs, both cell line-derived and patient-derived, being substantially more sensitive than non-malignant accessory cells from MM patients or HDs.

### QNZ alters cell cycle progression, induces mitochondrial dysfunction, and promotes apoptosis in MM cells

**Figure 2:**
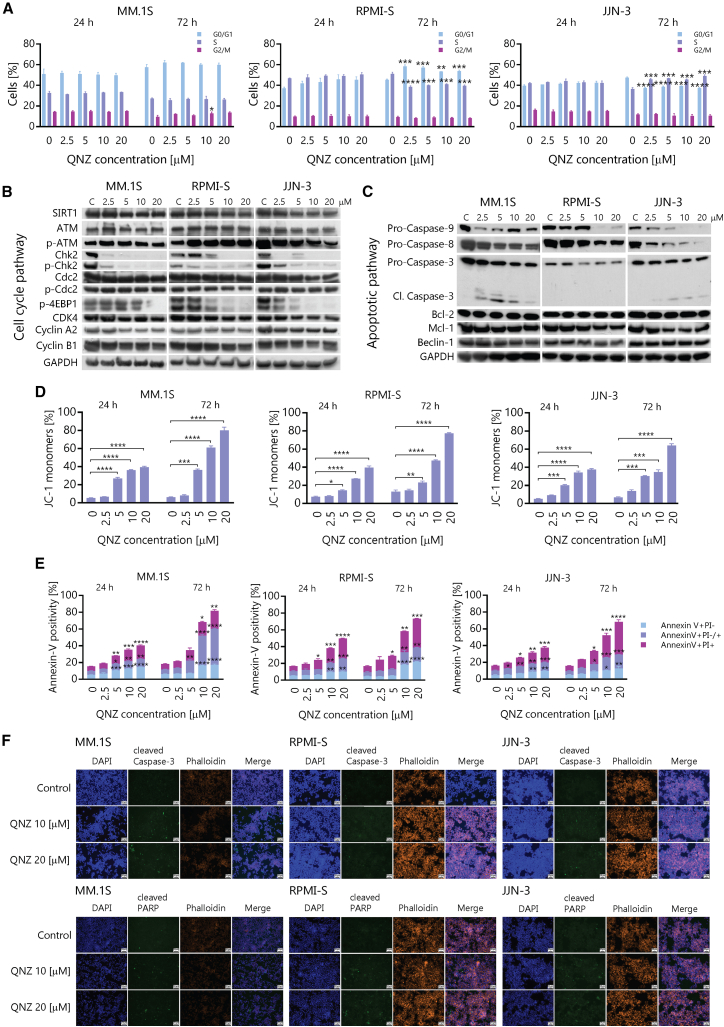
QNZ effects on cell cycle, apoptosis, and mitochondrial membrane potential in MM cells (A) Cell cycle distribution (G_0_/G_1_, S, and G_2_/M phases) in MM.1S, RPMI-S, and JJN-3 cells treated with QNZ (2.5, 5, 10, and 20 μM) for 24 and 72 h. Data represent the mean ± SD. Statistical significance versus control: ∗*p* < 0.05; ∗∗*p* < 0.01; ∗∗∗*p* < 0.001; ∗∗∗∗*p* < 0.0001 (one-way ANOVA, Dunnett’s test). (B) Cell cycle- and (C) apoptotic-related protein expression in MM cells after 72 h QNZ exposure by western blot. Proteins shown: SIRT1, ATM, *p*-ATM, Chk2, *p*-Chk2, Cdc2, *p*-Cdc2, p-4EBP1, CDK4, cyclin A2, cyclin B1, Pro-caspase-9, Pro-caspase-8, Pro-caspase-3, Bcl-2, Mcl-1, Beclin-1, and GAPDH (serves as a loading control) antibodies. Data are representative of two independent experiments. (D) Mitochondrial membrane potential (ΔΨm) in QNZ-treated MM cells increased JC-1 monomer signal (green) relative to JC-1 aggregate signal (red) indicates depolarization. (E) Apoptotic populations in QNZ-treated MM cells determined by Annexin V-FITC/PI dual staining: Annexin V^+^/PI^−^ (early apoptosis), Annexin V^+^/PI^−/+^ (late apoptosis), and Annexin V^+^/PI^+^ (necrosis). Data represent mean ± SD. Statistical comparisons versus control: ∗*p* < 0.05, ∗∗*p* < 0.01, ∗∗∗*p* < 0.001, and ∗∗∗∗*p* < 0.0001 (one-way ANOVA, Dunnett’s test). (F) Immunofluorescence of cleaved caspase-3 (first image) and cleaved PARP (second image) in MM.1S, RPMI-S, and JJN-3 cells (left to right) treated with vehicle or QNZ (10 or 20 μM). Each panel shows DAPI (blue), target protein (green), F-actin/phalloidin (orange), and merged channels.

To investigate the molecular mechanisms underlying QNZ’s anti-myeloma activity, MM.1S, RPMI-S, and JJN-3 cells were treated with 2.5, 5, 10, or 20 μM QNZ for 24 and 72 h. Initially, to characterize how escalating doses of QNZ influence proliferative dynamics in MM cell lines, we quantitatively assessed the cell-cycle profiles. At 72 h, RPMI-S cells exhibited a dose-dependent accumulation in G_0_/G_1_ phase, whereas JJN-3 cells showed an increase in S phase fraction; MM.1S cells remained largely unaffected, and none of the lines displayed significant alterations at 24 h (Figure 2A). Concordantly, immunoblotting revealed downregulation of key cell-cycle regulators, such as SIRT1 (predominantly in RPMI-S and JJN-3 cells), Chk2 and *p*-Chk2, Cdc2 (mostly JJN-3 cells), *p*-Cdc2 (mostly RPMI-S), p-4EBP1, CDK4, cyclin A2 (primarily MM.1S cells and JJN-3 cells), and cyclin B1. Phospho-ATM levels decreased in JJN-3 but increased in RPMI-S cells, while total ATM remained largely unchanged, except in JJN-3 cells treated with 10–20 μM QNZ (Figures 2B and S1).

Loss of mitochondrial membrane potential (ΔΨm), an early apoptotic hallmark, was assessed by JC-1 staining and flow cytometry. QNZ induced a concentration-dependent rise in JC-1 monomers, indicating ΔΨm collapse, beginning at 5 μM in all three MM cell lines at both 2 h and 72 h (Figure 2D). Annexin-V/propidium iodide (PI) assays confirmed that QNZ (5–20 μM) elicited a pronounced, dose-dependent increase in early and late apoptosis, followed by necrosis, with the magnitude of response greatest in MM.1S, then RPMI-S, and least in JJN-3 cells (Figure 2E). Mechanistically, immunoblotting demonstrated depletion of pro-caspase-9, -8, and -3 along with downregulation of anti-apoptotic Mcl-1 (primarily RPMI-S and JJN-3 cells), whereas levels of Bcl-2 (except in RPMI-S cells treated with 20 μM QNZ) and Beclin-1 remained unaltered, consistent with a mitochondrial- and caspase-dependent apoptotic cascade (Figures 2C and S1). Furthermore, immunofluorescence staining of apoptotic effectors was performed on MM.1S, RPMI-S, and JJN-3 cells treated with vehicle (control), 10, or 20 μM QNZ. After 72 h, a clear concentration-dependent increase in apoptotic labeling was observed: immunoreactivity for cleaved caspase-3 and cleaved PARP progressively increased with higher QNZ doses in all three cell lines. Quantitative image analysis confirmed these dose-dependent elevations in both markers (Figure 2F). Collectively, these results indicate that QNZ exerts potent anti-myeloma activity through a multifaceted mechanism: it induces G_0_/G_1_ or S-phase arrest in a cell-context-dependent manner and disrupts mitochondrial integrity to activate caspase- and PARP-mediated apoptosis.

### QNZ abrogates NF-κB signaling and induces transcriptomic reprogramming in MM cells

**Figure 3:**
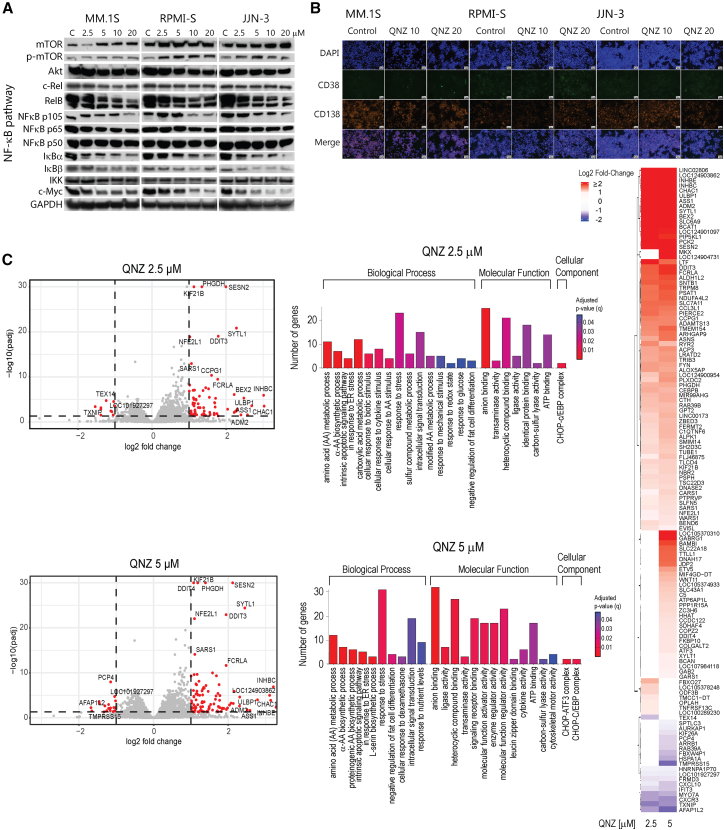
QNZ effects on NF-κB signaling and transcriptomic profiles in MM cells (A) NF-κB pathway protein expression in MM.1S, RPMI-S, and JJN-3 cells after 72 h QNZ exposure by western blot. Proteins shown: mTOR, *p*-mTOR, Akt, c-Rel, RelB, NF-κB p105, NF-κB p65, NF-κB p50, IκBα, IκBβ, IKK, and c-Myc. GAPDH serves as loading control. Blots are representative of two independent experiments. (B) Immunofluorescence of CD38 and CD138 in MM.1S, RPMI-S, and JJN-3 cells (left to right) treated with vehicle or QNZ (10 or 20 μM). Columns represent treatment conditions; rows correspond to DAPI (blue, nuclei), CD38 (green), CD138 (orange), and merged channels. (C) Volcano plots showing differentially expressed genes in MM.1S, RPMI-S, and JJN-3 cells treated with 2.5 or 5 μM QNZ versus untreated controls. The *x* axis represents log_2_ fold change (log2FC) and the *y* axis represents −log_10_ adjusted *p* value; genes meeting significance thresholds (|log2FC| > 1; FDR-adjusted *p* ≤ 0.05) are highlighted in red, non-significant genes in gray. Gene Ontology (GO) enrichment bar graphs show significantly enriched biological process, molecular function, and cellular component terms for upregulated and downregulated gene sets at 2.5 and 5 μM QNZ in all three cell lines. Heatmap shows normalized expression of all significantly deregulated genes across MM.1S, RPMI-S, and JJN-3 cells treated with 2.5 or 5 μM QNZ.

To further elucidate QNZ’s effects on NF-κB signaling pathways, we assessed principal NF-κB subunits and their regulatory proteins. Importantly, QNZ attenuated NF-κB-driven survival signaling: protein expression of c-Rel (predominantly MM.1S and RPMI-S cells), RelB, NF-κB p105, NF-κB p65, and both IκBα and IκBβ isoforms was markedly reduced. In parallel, QNZ suppressed Akt (primarily RPMI-S and JJN-3 cells) and c-Myc expression yet paradoxically upregulated mTOR and its phosphorylated form. Notably, NF-κB p50 and IKK protein levels remained unchanged (Figures 3A and S1). Immunofluorescence analysis of myeloma markers was performed on MM.1S, RPMI-S, and JJN-3 cells treated with vehicle (control), 10, or 20 μM QNZ. Assessment of canonical myeloma surface markers demonstrated that CD38 and CD138 expression and subcellular localization remained unchanged at 24 h post-treatment in all three cell lines, with no detectable alteration in fluorescence intensity or membrane distribution across conditions (Figure 3B).

Transcriptomic profiling was performed to characterize QNZ-induced gene expression alterations across MM cell lines. Applying a significance threshold of false discovery rate (FDR) <0.05 and log2FC > 1 and log2FC < −1 identified 76 upregulated and 10 downregulated genes at 2.5 μM QNZ, and 100 upregulated and 18 downregulated genes at 5 μM QNZ, that were commonly deregulated in MM.1S, RPMI-S, and JJN-3 cells (Figure 3C). The extent of transcriptional deregulation was visualized by volcano plots, which revealed a broad distribution of significantly modulated transcripts. Among the most significantly upregulated genes shared across all three MM cell lines following both 2.5 and 5 μM, QNZ treatment were KIF21B, PHGDH, SESN2, NFE2L1, DDIT3, SYTL1, SARS1, FCRLA, INHBC, ULBP1, CHAC1, ASS1, and ADM2. Distinct concentration-dependent upregulation was observed for CCPG1 and BEX2 at 2.5 μM QNZ and for DDIT4 and INHBE at 5 μM QNZ. Conversely, TXNIP and TEX14 showed significant downregulation at 2.5 μM QNZ, whereas PCP4, AFAP1L2, and TMPRSS15 were selectively repressed at 5 μM QNZ. The corresponding heatmap illustrates the expression patterns of all deregulated genes in both QNZ concentrations, consistently across the three MM lines. Functional enrichment analysis revealed that QNZ treatment modulated multiple biological processes and molecular functions. Commonly deregulated pathways in all three MM cell lines included biological processes related to amino acid and carboxylic acid metabolism, cellular response to stress, and intracellular signal transduction. Enriched molecular functions were associated with anion binding, heterocyclic compound binding, identical protein binding, and ATP binding. Additional shared processes included the α-amino acid biosynthetic process, the intrinsic apoptotic signaling pathway in response to ER stress, and the negative regulation of adipocyte differentiation. Consistently, molecular functions such as transaminase, ligase, and carbon-sulfur lyase activities, as well as cellular components involving the CHOP-C/EBP complex, were significantly affected. In addition, transcriptomic profiling was performed on primary MM patient samples (pt1–pt4) treated with QNZ. Prinicipal-component analysis (PCA) demonstrated a clear separation between QNZ-treated and control samples along PC1, confirming both inter-patient heterogeneity and a transcriptional response to QNZ (Figure S2A). Analysis of differentially expressed genes identified transcripts commonly deregulated across all four, or at least three, patient samples, including prominently downregulated genes such as SAP25, ZNF75D, GCLM, AMN1, SLC7A11, and HPS3 (Figure S2B). Gene Ontology (GO) enrichment analysis of these modulated genes revealed significant perturbation of biological processes related to nucleic acid and macromolecule metabolism, chromatin organization, and cellular response to stress, alongside molecular functions encompassing nucleic acid binding, glutamate-cysteine ligase activity, and zinc ion transport (Figure S2C). A comprehensive list of QNZ-regulated genes in MM cell lines and primary patient samples is provided in Table S2. Together, these findings indicate that QNZ dismantles pro-survival NF-κB/Akt/c-Myc signaling, modulates mTOR activity, and enforces a broad, stress-associated metabolic and apoptotic transcriptional reprogramming in MM cells.

### QNZ exerts anti-proliferative and cytotoxic effects on MM cells, with partial protection conferred by bone marrow stromal cells

**Figure 4:**
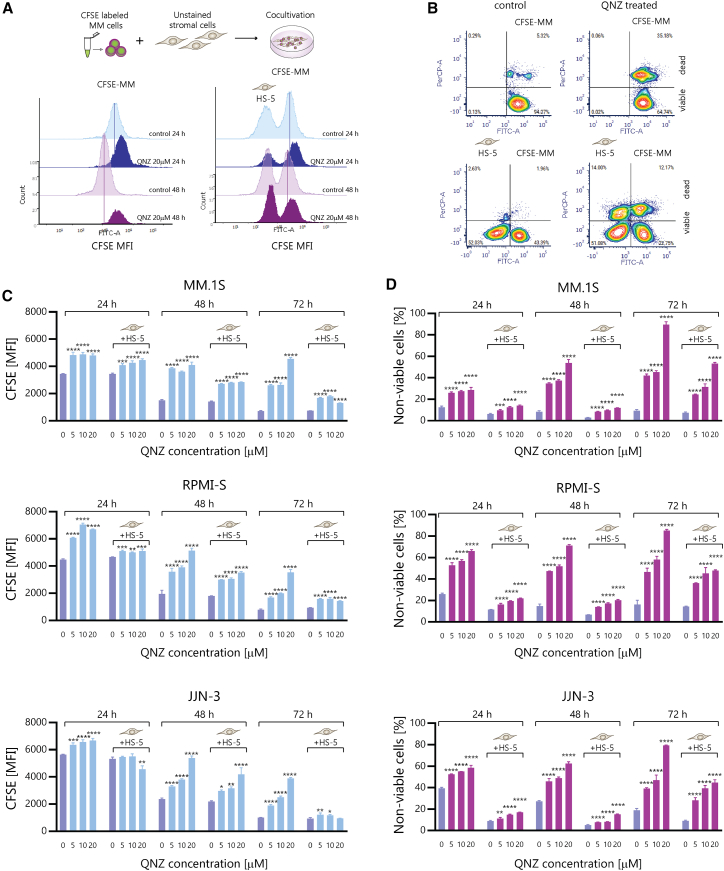
Effect of QNZ on MM.1S cell proliferation and cytotoxicity in monoculture and stromal co-culture CFSE-labeled MM cells were cultured alone or with unlabeled HS-5 bone marrow stromal cells and treated with QNZ (5, 10, or 20 μM) for 24, 48, or 72 h. Proliferation was assessed by CFSE dilution, and cytotoxicity by 7-AAD uptake in MM cells (CFSE). (A) Representative CFSE fluorescence histograms for MM.1S monocultures (left) and MM.1S + HS-5 co-cultures (right) at 24 and 48 h, showing untreated controls and QNZ-treated samples (20 μM). (B) Representative CFSE *vs.* 7-AAD dot plots for MM.1S monocultures (top row) and MM.1S + HS-5 co-cultures (bottom row), for untreated controls (left) and QNZ-treated samples (right). (C) Bar graph depicting CFSE median fluorescence intensity in viable MM cells (CFSE^+^/7-AAD^−^) after 24, 48, and 72 h of treatment with 5, 10, and 20 μM QNZ, in monoculture and co-culture. (D) Bar graph showing the percentage of non-viable MM cells (CFSE^+^/7-AAD^+^) at 24, 48, and 72 h at QNZ concentrations (5, 10, and 20 μM), in monoculture and co-culture. Data are presented as mean ± SD. Statistical significance *versus* control: ∗*p* < 0.05; ∗∗*p* < 0.01; ∗∗∗*p* < 0.001; ∗∗∗∗*p* < 0.0001 (one-way ANOVA, Dunnett’s test).

To determine whether the cytotoxic effect of QNZ on MM cells is modulated by the presence of bone marrow stromal cells, we compared the proliferation of MM cells cultured alone versus in direct co-culture with HS-5 stromal cells. MM cells were labeled with carboxyfluorescein diacetate succinimidyl ester (CFSE) and then either maintained in monoculture or seeded onto unlabeled HS-5 monolayers. After treatment with QNZ (20 μM) or vehicle control, MM cell division was evaluated by flow cytometric analysis of CFSE fluorescence; proliferating cells exhibit successive halving of CFSE intensity, yielding a characteristic histogram of viable, CFSE-positive MM cells distinct from CFSE-negative HS-5 cells (Figure 4A). Viability was assessed by 7-aminoactinomycin D (7-AAD) staining, and representative dot plots depict viable MM cells in monoculture (top row) versus co-culture with HS-5 stromal cells (bottom row) after QNZ treatment (right dot plots), which show an increase in cell death compared to vehicle-treated controls (left dot plots; Figure 4B).

CFSE-labeled MM cells were cultured with increasing concentrations of QNZ (5–20 μM) in the presence or absence of HS-5 bone marrow stromal cells for 24, 48, and 72 h. Cell proliferation of MM cell lines (MM.1S, RPMI-S, and JJN-3 cells) was significantly inhibited by QNZ in a dose- and time-dependent manner in both monoculture and co-culture conditions. Increased CFSE signal corresponded to decreased proliferation. However, the antiproliferative effect of QNZ was slightly less pronounced in the presence of stromal cells, with the most notable differences observed at 72 h (Figure 4C). Similarly, QNZ treatment induced a significant, concentration- and time-dependent increase in the proportion of non-viable MM cells across all tested MM lines, both in monoculture and in co-culture with HS-5 stromal cells. Stromal co-culture conferred a partial cytoprotective effect, attenuating QNZ-induced cytotoxicity, most prominently at 24 and 48 h, although this protective effect diminished slightly by 72 h (Figure 4D). In summary, QNZ exerts potent antiproliferative and cytotoxic effects on MM cells in a dose- and time-dependent manner, with bone marrow stromal cells partially mitigating these effects.

### QNZ reduces tumor burden *in vivo*

**Figure 5:**
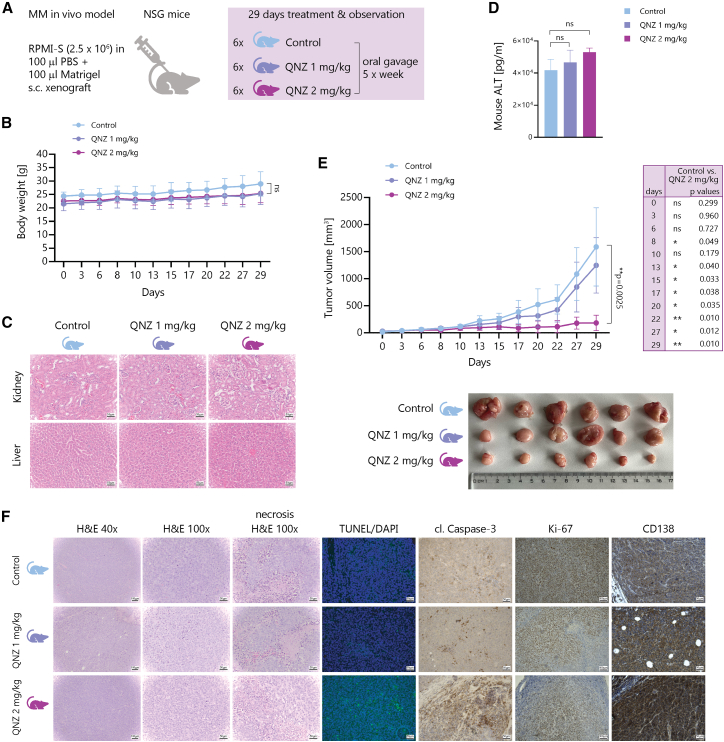
QNZ effects on MM xenograft tumor growth and tolerability *in vivo* (A) Schematic of the *in vivo* study design. NSG mice bearing subcutaneous RPMI-S xenografts were randomized into three groups (*n* = 6 per group) receiving daily oral gavage of vehicle, QNZ 1 mg/kg, or QNZ 2 mg/kg for 4 weeks (5 days on, 2 days off). (B) Mouse body weights across treatment groups over the study duration (ns = not significant). (C) Representative H&E images (100×) of kidneys (upper row) and livers (lower row) from vehicle-, QNZ 1 mg/kg-, and QNZ 2 mg/kg-treated mice at study endpoint. (D) Plasma ALT concentrations at study endpoint in vehicle-, QNZ 1 mg/kg-, and QNZ 2 mg/kg-treated mice. (E) Tumor growth curves in NSG mice bearing subcutaneous RPMI-S xenografts treated with vehicle or QNZ (1 or 2 mg/kg; *n* = 6 per group). Tumor volumes at study endpoint with representative images of excised tumors from vehicle- (top row), QNZ 1 mg/kg- (middle row), and QNZ 2 mg/kg-treated mice (bottom row). Data are presented as mean ± SD. Statistical significance: ∗*p* < 0.05; ∗∗*p* < 0.01 (two-way ANOVA, Tukey’s test). (F) Tumor histology and immunostaining images arranged by treatment group from top to bottom (control, QNZ 1 mg/kg, QNZ 2 mg/kg) and stain/marker from left to right: H&E at 40× (column 1), H&E at 100× (column 2), H&E at 100× showing necrotic regions (column 3), TUNEL/DAPI (column 4), cleaved caspase-3 immunohistochemistry (column 5), Ki-67 immunohistochemistry (column 6), and CD138 immunohistochemistry (column 7). Scale bars and magnifications are indicated on individual images.

To assess the *in vivo* efficacy of QNZ against MM, NSG mice were subcutaneously inoculated with RPMI-S cells and, once tumors were established, randomized to receive daily oral gavage of vehicle, 1 mg/kg QNZ, or 2 mg/kg QNZ (5 days on/2 days off) for four consecutive weeks (*n* = 6 per group; Figure 5A). Treatment was well tolerated, as evidenced by stable body weights across all cohorts (Figure 5B). Further histopathological examination of harvested livers and kidneys revealed no treatment-related abnormalities: renal cortico-medullary architecture and liver lobular architecture were preserved across control and QNZ-treated mice, with only minimal, focal changes observed in ≤5% of renal tubules or glomeruli (Figure 5C). QNZ treatment did not result in a statistically significant change in plasma alanine aminotransferase (ALT) levels (Figure 5D). Administration of 2 mg/kg QNZ resulted in a significant reduction in tumor volume, with effects becoming evident by day 13 and persisting throughout the study (*p* = 0.0025 vs. vehicle). End-of-study images illustrate visibly smaller tumors in 2 mg/kg QNZ group relative to both 1 mg/kg QNZ and vehicle groups (Figure 5E). Hematoxylin and eosin (H&E) staining showed that tumors in all groups were massive, diffuse, and infiltrative, with high mitotic activity and frequent confluent necrosis, and overall architecture was largely preserved across treatments. Quantitative immunohistochemistry (IHC) and TUNEL analysis revealed dose-dependent increases in apoptotic markers: TUNEL positivity was ∼1%–3% (control), ∼5%–10% (QNZ 1 mg/kg) and ∼15%–20% (QNZ 2 mg/kg). Cleaved caspase-3 staining was observed across all groups and increased with treatment (control: ∼1%–5%; QNZ 1 mg/kg: ∼5%–15%; QNZ 2 mg/kg: ∼15%–30%), whereas Ki-67 indices indicated high proliferative activity in controls (∼90%–98%) with a modest reduction in the QNZ 1 mg/kg group (∼70%–90%) and a marked decrease in the QNZ 2 mg/kg group (∼30%–50%). CD138 staining remained strongly positive in all tumors, confirming maintenance of the plasma cell phenotype (Figure 5F). Collectively, these findings indicate that QNZ produces dose-dependent antitumor effects in this MM xenograft model: the 2 mg/kg regimen produced the largest and most sustained tumor-volume reduction and the greatest induction of apoptosis and reduction in proliferative regions, while overall malignant architecture and CD138 expression were preserved.

### Synergistic cytotoxicity of QNZ combined with anti-MM agents

**Figure 6:**
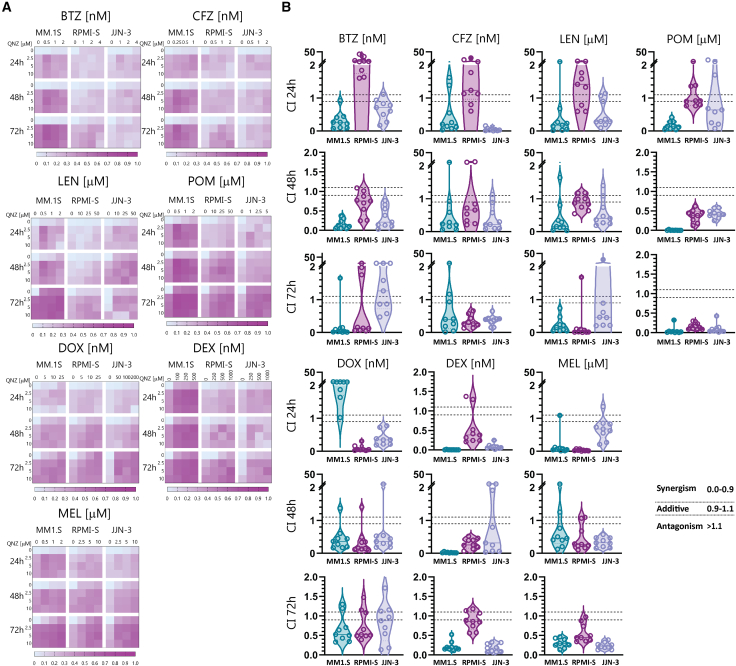
QNZ and anti-MM drug combination effects on MM cell viability (A) Heatmaps display the fraction affected (Fa; proportion of nonviable cells) for QNZ alone, each anti-MM agent alone (bortezomib [BTZ], carfilzomib [CFZ], lenalidomide [LEN], pomalidomide [POM], doxorubicin [DOX], dexamethasone [DEX], and melphalan [MEL]), and their combinations in MM.1S, RPMI-S, and JJN-3 cells. (B) Combination indices (CI) derived by the Chou-Talalay method are plotted against Fa; CI < 1, = 1, and >1 denote synergism, additivity, and antagonism, respectively. Data are presented as median ± IQR.

Co-administration of next-generation and conventional anti-MM therapies enhances clinical efficacy compared to single-agent treatment. To explore this synergistic potential, we evaluated combinations of QNZ with novel and established anti-MM agents. We assessed the anti-neoplastic synergy of QNZ with next-generation proteasome inhibitors (BTZ, CFZ), IMiDs (LEN, POM), and conventional chemotherapeutics (DEX, DOX, MEL) in MM cell lines (MM.1S, RPMI-S, and JJN-3). Cell viability was quantified by MTT assay at 24, 48, and 72 h post-treatment. Synergistic interactions were evaluated by generating heatmaps of the fraction affected (Fa) for each drug pairing (Figure 6A), and combination indices (CIs) were calculated using CalcuSyn software according to the Chou-Talalay method. The resulting isobolograms illustrate CI values for each combination relative to the corresponding single-agent treatments (Figure 6B).

The first-generation proteasome inhibitor BTZ exhibited pronounced synergism with QNZ in MM.1S cells at 24, 48, and 72 h. In RPMI-S cells, BTZ combined with QNZ was initially antagonistic at 24 h, shifted to predominantly synergistic interactions at 48 h, and achieved even stronger synergy by 72 h. Conversely, JJN-3 cells displayed the opposite temporal pattern: predominantly synergistic effects at 24 h, strong synergy at 48 h, and predominantly antagonistic effects by 72 h. Likewise, the second-generation proteasome inhibitor CFZ demonstrated mostly synergistic cytotoxicity when administered alongside QNZ in MM.1S cells across all time points. In RPMI-S cells, the CFZ-QNZ combination transitioned from antagonism at 24 h to moderate synergy at 48 h and strong synergy at 72 h. By contrast, JJN-3 cells exhibited robust synergy with CFZ-QNZ at all time points. IMiD combinations followed analogous trends. LEN combined with QNZ induced significant synergism in MM.1S and JJN-3 cells at all time points, whereas RPMI-S cells displayed antagonism at 24 and 48 h before converting to strong synergy at 72 h. POM, a second-generation IMiD, combined with QNZ yielded consistently strong synergy in MM.1S and JJN-3 cells over 24–72 h; in RPMI-S cells, the interaction was antagonistic at 24 h but became synergistic at 48 and 72 h.

Doxorubicin (DOX), a conventional anti-MM chemotherapeutic, exhibited antagonistic interactions with QNZ in MM.1S cells at 24 h, which converted to synergistic cytotoxicity at 48 and 72 h. In RPMI-S and JJN-3 cells, DOX in combination with QNZ was synergistic at all time points, although in JJN-3 cells, the effect at 72 h ranged from additive to synergistic. Similarly, QNZ in combination with DEX or MEL elicited predominantly synergistic effects over 24, 48, and 72 h. The QNZ-DEX pairing achieved its greatest synergy in MM.1S cells, whereas QNZ in combination with MEL exhibited maximal synergism in RPMI-S cells. Overall, significant synergistic effects of QNZ were observed when combined with both proteasome inhibitors and IMiDs, showing more potent effects with second-generation agents such as CFZ and POM. In addition, QNZ demonstrated synergy with conventional drugs, particularly DEX and MEL, suggesting strong potential for further investigation of these combinations in future clinical studies.

## Discussion

NF-κB is a family of transcription factors that orchestrates inflammation, proliferation, and survival. Its constitutive activation in cancer drives proliferation, resistance to apoptosis, angiogenesis, EMT, metastasis, and therapy resistance, reshaping the tumor microenvironment and making NF-κB a high-value but complex therapeutic target.^16^ In MM, canonical NF-κB signaling is chronically fueled by bone marrow cytokines (e.g., TNF), which, via adaptor proteins such as TRAFs, activate the IKK complex (IKKα, IKKβ, and NEMO) to phosphorylate IκBs (mainly IκBα), triggering their ubiquitin-dependent proteasomal degradation. Released NF-κB dimers (predominantly p50-RelA and p50-c-Rel) translocate to the nucleus to drive transcription of pro-survival and inflammatory programs that sustain the malignant phenotype.^2^ The noncanonical NF-κB pathway, triggered by select TNFRSF members (e.g., CD40, BAFFR, LTβR, RANK), is NEMO-independent and driven by stabilization of NIK, which is frequently activated by NIK-stabilizing mutations. NIK activates IKKα homodimers that phosphorylate p100, promoting its partial proteasomal processing to p52; p52-RelB dimers then translocate to the nucleus to reprogram transcription toward survival and chemoresistance.^17^ Crosstalk can produce sustained RelB:p50 activity linked to poor treatment response, arguing for combined inhibition of both NF-κB arms as a therapeutic strategy in MM.^1^

Canonical NF-κB subunits (NF-κB p105, NF-κB p65, and NF-κB p50), RelB, and IκBα were broadly expressed across the ten MM cell lines, but their activation state was heterogeneous. Increased p-NF-κB (p65) was observed in MM.1S, OPM-1, RPMI-S, JJN3, and KMS11 cells. Phospho-IκBα was prominent in MM.1S and RPMI-S cells, detectable in JJN3 cells, and weaker in several DOX-resistant RPMI derivatives (RPMI-DOX6, RPMI-DOX40, RPMI-LR5, RPMI-MR20 cells), indicating increased canonical NF-κB activity in those lines. c-Rel expression was largely restricted to OPM-1 cells, with only faint signals in MM.1S, RPMI-S, and RPMI-MR20 cells, suggesting a cell line-specific contribution of this subunit to NF-κB transcriptional programs.

QNZ, a quinazoline derivative, potently inhibits NF-κB activation and nuclear translocation, suppressing expression of pro-survival and pro-inflammatory genes and thereby impairing proliferation, migration, and survival of tumor-promoting cell.^9^ Our data show that QNZ markedly suppressed NF-κB-driven survival signaling: c-Rel, RelB, NF-κB p105, NF-κB p65, and IκBα/β were reduced, with concurrent downregulation of Akt and c-Myc, consistent with loss of pro-survival and proliferative signals. Paradoxically, mTOR and phospho-mTOR were upregulated, suggesting compensatory mTOR activation despite NF-κB/Akt suppression. The lack of change in NF-κB p50, and IKK points to selective targeting within the NF-κB pathway. Overall, NF-κB subunits were abundant in MM but showed heterogeneous activation, and the NF-κB inhibitor QNZ reduced NF-κB-dependent survival signaling, suggesting that targeting NF-κB can impair survival of MM cells with high NF-κB activity.

QNZ consistently suppresses NF-κB-driven programs that promote tumor progression, angiogenesis, and metastasis across multiple preclinical models. Mechanistic studies show that QNZ decreases NF-κB activation and its downstream effectors (VEGF, TNF-α, IL-1β, IL-6, MMP-2/9, XIAP, cyclin D1), thereby attenuating cell invasion, migration, proliferation, metastatic potential, and angiogenic signaling in breast, hepatocellular, bladder, prostate, and non-small cell lung cancer models.^18^^,^^19^^,^^20^^,^^21^^,^^22^ In colorectal cancer, QNZ counteracts SERPINB5-mediated induction of phosphorylated p65, TNF-α, EMT markers, and VEGFA, reducing migratory, invasive, and proliferative phenotypes.^23^ Pathway-inhibitor profiling, including QNZ, identified a dominant role for JNK1/2 in PGE_2_-driven upregulation of uPA and MMP-9 and for Akt/ERK1/2 in PGE_2_-induced COX-2 expression and motility, all of which are susceptible to 17β-estradiol-mediated inhibition in LoVo colon carcinoma models.^24^^,^^25^^,^^26^ By contrast, in oxaliplatin-resistant LoVo cells, QNZ directly reduced ABCG2 and phospho-NF-κB levels while inducing endoplasmic-reticulum stress markers, thereby attenuating mechanisms of chemoresistance.^27^ Importantly, QNZ’s inhibitory effects extend to *in vivo* disease modulation: treatment reduced NF-κB and TNF-α expression, serum AFP, and liver nodule formation in chemical models of hepatocarcinogenesis, and decreased susceptibility to DEN-induced tumorigenesis in genetically sensitized mice.^14^^,^^28^ Beyond cancer cell-intrinsic processes, QNZ also mitigates non-malignant pathology and immune dysregulation, ameliorating 6-PPD-induced hepatic lipid accumulation, oxidative stress, and immune suppression in zebrafish (implicating PPARγ/NF-κB crosstalk) and impairing dendritic cell maturation and Th2 polarization by reducing MHCII/CD86 expression and pro-Th2 cytokine release.^28^^,^^29^ Collectively, these data position NF-κB blockade with QNZ as a multifaceted intervention that suppresses inflammation, angiogenesis, invasion, and genomic stress responses while modulating the immune milieu, but its pronounced context dependence necessitates defining therapeutic windows and tailoring rational combination strategies to exploit NF-κB-driven resistance pathways.

QNZ produced potent, time- and dose-dependent *in vitro* cytotoxicity across a ten-cell-line MM panel, with EC_50_ values decreasing from 8.8–44.8 μM at 24 h to 2.9–25.1 μM at 48 h and 1.5–6.3 μM at 72 h, corresponding to an approximately 3-fold and 7-fold increase in potency at 48 and 72 h versus 24 h; however, RPMI-DOX6, RPMI-DOX40, and KMS-11 exhibited relative resistance. Importantly, *ex vivo* primary bone-marrow-derived malignant PCs from MM patients (*n* = 15) were robustly sensitive to QNZ at 48 h (EC_50_ range 0.4–33.4 μM), whereas non-plasma cell mononuclear fractions from the same patients displayed markedly lower sensitivity (10/15 samples failed to reach 50% inhibition at 20 μM). Peripheral blood MNCs from HDs (*n* = 6) were essentially spared, with no sample reaching 50% growth inhibition at concentrations up to 40 μM. Prior work has reported low-micromolar activity of QNZ in MM monocultures and even nanomolar efficacy in sensitized models (e.g., LGR4-overexpressing cells), with modest attenuation of activity in stromal co-culture and reduced MM adhesion to stromal cells and fibronectin.^30^ In our study, QNZ retained potent, dose- and time-dependent antiproliferative and cytotoxic activity against MM cells in both monoculture and stromal co-culture. Bone marrow stromal cells afforded only partial, transient protection, most evident at earlier time points, whereas QNZ’s effects largely persisted over time. Mechanistically, NF-κB blockade is expected to disrupt pro-survival cytokine networks (e.g., TNF-α, IL-6) and macrophage-driven resistance pathways (as exemplified by IKK-16 studies), providing a plausible basis for the partial abrogation of stromal protection.^31^ In our *in vivo* study, daily oral QNZ (1 or 2 mg/kg, 5 days on/2 days off for 4 weeks) was well tolerated in NSG xenograf-bearing mice and did not produce body-weight loss. Histopathological analysis and terminal plasma ALT measurements indicate that daily oral QNZ at 1 and 2 mg/kg for 4 weeks did not cause overt hepatic or renal toxicity in this mouse model. The minimal, focal renal changes (≤5%), together with preserved hepatic architecture and non-significantly altered ALT, are most consistent with background or compensatory/regressive alterations rather than drug-induced injury, supporting a favorable short-term safety profile at the tested doses.

Treatment with 2 mg/kg QNZ produced a significant, dose-dependent suppression of tumor growth that became apparent by day 13 and persisted through the end of the study (*p* = 0.0025 versus vehicle), with visibly smaller tumors observed at necropsy. Histopathological and IHC analyses suggest that QNZ exerts its antitumor effects *in vivo* primarily through induction of apoptosis, most prominently at the 2 mg/kg dose, with a reduction in proliferative activity observed in a subset of tumors. The persistence of a CD138-positive plasma cell phenotype indicates that QNZ does not induce phenotypic loss of MM identity. Collectively, these analyses demonstrate that QNZ treatment is associated with increased apoptotic cell death and decreased proliferative activity, providing mechanistic evidence supporting its antitumor activity *in vivo*. These *in vivo* antitumor effects are consistent with prior preclinical reports in other tumor settings: in a chemically induced, inflammation-driven rat model of hepatocellular carcinoma, QNZ markedly reduced tumor burden and liver inflammation while suppressing NF-κB activation, TNF-α/IL-6 levels, and proliferative/angiogenic markers.^14^ In uveal melanoma xenografts with acquired resistance to BET inhibition (PLX51107), co-treatment with QNZ reversed resistance and produced superior tumor suppression relative to either agent alone, accompanied by reduced p65 phosphorylation, lowered CEBPD expression, and PARP-cleavage-mediated apoptosis, without detectable host toxicity.^32^ Similarly, combined QNZ and AKT inhibition cooperatively suppressed NF-κB signaling across xenograft and syngeneic models, increased apoptosis, downregulated NF-κB target genes and anti-apoptotic genes, and achieved greater tumor reduction than monotherapy.^8^ Statistical analyses of our *in vitro* and *in vivo* data demonstrate that QNZ selectively targets malignant PCs, both cell line-derived and patient-derived, while largely sparing non-malignant bone marrow accessory cells and HD peripheral MNCs, indicating a favorable therapeutic window. These concordant findings support further mechanistic investigation, dose optimization and combinatorial evaluation of QNZ in microenvironment-competent myeloma models.

Across diverse tumor models, pharmacological NF-κB inhibition with QNZ consistently attenuates pro-survival and pro-invasive phenotypes, although its effects are strikingly context-dependent. QNZ reduced anti-apoptotic protein expression and invasive behavior in non-small cell lung cancer and potently induced both extrinsic and intrinsic apoptosis in hepatocellular carcinoma cells.^33^^,^^34^ As a mechanistic probe, QNZ showed that blockade of ERK/NF-κB signaling mediates the antiglioblastoma activity of amentoflavone and, together with other NF-κB inhibitors, modulates proliferation and inflammatory signaling and sensitizes head-and-neck squamous carcinoma cells to FasL-induced extrinsic apoptosis with enhanced caspase-8/-3 activation.^35^^,^^36^ In cervical carcinoma models, QNZ reduces cleavage of caspase-12 and caspase-3 and, by inhibiting NF-κB during ER stress (e.g., brefeldin, BFA), upregulates CHOP and shifts cells toward caspase-independent, CHOP-mediated apoptosis despite transient autophagy suppression.^37^ In colorectal and pancreatic models, QNZ suppressed pro-motility/pro-invasive NF-κB signaling (including TRAIL-DR4/DR5 and IκBα/p65 dynamics), increased p65/IκBα phosphorylation, upregulated Bax, and downregulated Bcl-2 and MMP2, indicating NF-κB-dependent antiproliferative and proapoptotic effects.^38^^,^^39^ In this study, QNZ induces mitochondrial dysfunction in MM cells, with loss of mitochondrial membrane potential observed at ≥5 μM after 24 and 72 h. Cell-death assays using phosphatidylserine/PI staining showed a dose-dependent increase in early and late apoptosis that progressed to secondary necrosis, with sensitivity ranked MM.1S > RPMI-S > JJN-3 cells. Analysis of apoptosis-regulatory proteins further confirmed activation of a caspase-dependent intrinsic apoptotic program, characterized by depletion of pro-caspase-9, -8, and -3 and downregulation of Mcl-1, while Bcl-2 and Beclin-1 remained unchanged, a profile consistent with mitochondria-mediated, caspase-dependent cell death. Retention of CD38 and CD138 at 24 h, despite robust apoptotic signaling, suggests that QNZ elicits a time-dependent, executioner caspase-dependent apoptotic program, beginning with phosphatidylserine externalization (Annexin-PE positivity) and culminating in caspase-3 activation and PARP cleavage. This temporal dissociation supports a model in which QNZ rapidly engages upstream death signals that converge on executioner caspases, driving proteolytic dismantling of nuclear and cytoplasmic substrates prior to detectable downregulation of lineage-defining surface antigens or overt membrane remodeling.

Beyond promoting apoptosis and inhibiting invasion, QNZ impairs DNA-repair responses, for example, it reduces hydroquinone-induced homologous recombination in osteosarcoma, and provides a quinazoline scaffold for derivatives (e.g., QNZ-A) that induce ROS-dependent G_2_/M arrest and cell death in lung cancer.^15^^,^^40^ QNZ also suppresses cell-cycle progression in MM: in LGR4-overexpressing MM models it decreased the S-phase fraction, consistent with cell-cycle arrest.^30^ In osteosarcoma, QNZ downregulated cyclin D1, CDK6, and cyclin A, key regulators of the G_1_/S and G_2_/M transitions, yielding G_1_ arrest and reduced proliferation.^41^ Our cell-cycle analyses indicate that QNZ exerts time- and cell line-dependent antiproliferative effects: significant cell-cycle redistribution was apparent at 72 h (RPMI-S: G_0_/G_1_ accumulation; JJN-3: increased S-phase fraction), whereas MM.1S remained largely unresponsive. Immunoblotting revealed coordinated downregulation of cell-cycle regulators (SIRT1, Chk2/p-Chk2, Cdc2/p-Cdc2, CDK4, cyclins A2, and B1) and reduced p-4EBP1, consistent with impaired cell-cycle progression and diminished cap-dependent translation. The opposing regulation of phospho-ATM (increased in RPMI-S, decreased in JJN-3) and the relative stability of total ATM points to differential engagement of DNA damage/replication stress signaling across cell lines. Taken together, the G_0_/G_1_ accumulation in RPMI-S is consistent with activation of a checkpoint program linked to CDK4 and Cdc2 inhibition (and increased *p*-ATM), whereas the S-phase enrichment in JJN-3 may reflect impaired resolution of replication stress or defective checkpoint signaling (concomitant with reduced *p*-ATM and Chk2). The downregulation of SIRT1 and p-4EBP1 further suggests that QNZ’s effects modulate chromatin/repair regulators and cap-dependent translation, which may amplify antiproliferative responses. Overall, these findings reveal heterogeneous QNZ sensitivity and implicate checkpoint signaling, translational control, and context-dependent induction of caspase-dependent intrinsic apoptosis as determinants of response.

In MM, constitutive NF-κB signaling is a well-established driver of survival, proliferation, and drug resistance, and its pharmacological blockade is expected to elicit broad transcriptional reconfiguration. The consistent deregulation of nearly 86–118 genes across MM.1S, RPMI-S, and JJN-3 cells substantiates the robustness of QNZ’s transcriptional effects. NF-κB and ER stress signaling are bidirectionally interconnected: ER stress activates NF-κB via the IRE1α-IKK axis, while NF-κB normally represses pro-apoptotic ER stress gene programs, including DDIT3/CHO.^42^ QNZ-mediated NF-κB inhibition thus de-represses these stress-responsive programs, an effect well-reflected in the transcriptomic signature presented in our study. Among the most significantly and uniformly upregulated transcripts was DDIT3, encoding the pro-apoptotic transcription factor CHOP/GADD153, a canonical terminal effector of ER stress-induced apoptosis. Under conditions of sustained ER stress, CHOP orchestrates apoptosis through downregulation of BCL-2, upregulation of DR5/TNFRSF10B, and induction of BH3-only pro-apoptotic proteins, primarily via the PERK-IF2α-ATF4 UPR axis. MM cells are uniquely susceptible to ER stress-inducing agents given their constitutively high secretory activity, which keeps them near the threshold of proteostatic capacity. CHOP-dependent apoptosis has been previously demonstrated in myeloma via Activin A-Smad3 signaling, and its induction here is reinforced by the enrichment of CHOP-C/EBP (at both QNZ concentrations) and CHOP-ATF3 complexes (selectively at 5 μM), reflecting a dose-escalated commitment to terminal UPR signaling.^43^^,^^44^ The consistent upregulation of PHGDH, the rate-limiting enzyme of de novo serine biosynthesis, alongside pathway-level enrichment of amino acid, carboxylic acid, and L-serine biosynthetic processes, indicates a compensatory metabolic stress response aimed at restoring anabolic capacity and redox balance in QNZ-treated cells. The co-induction of ASS1 (argininosuccinate synthase 1), a key enzyme in the urea cycle whose upregulation has been linked to PERK/eIF2α/ATF4/CHOP pathway activation, further suggests a metabolic-ER stress crosstalk mechanism that amplifies the pro-apoptotic UPR program in QNZ-treated MM cells.^8^^,^^45^^,^^46^ The QNZ transcriptional signature reveals significant convergence on oxidative stress and redox regulatory networks. SESN2 (sestrin-2), consistently upregulated across all conditions, functions as a potent suppressor of ROS accumulation and a negative regulator of mTORC1 via AMPK activation, consistent with QNZ’s known capacity to inhibit mitochondrial complex I and suppress mTORC1 signaling. The concurrent upregulation of CHAC1, a γ-glutamyl cyclotransferase that degrades glutathione downstream of the ATF4-CHOP UPR arm, creates a positive feedback loop in which ER stress amplifies oxidative stress, a mechanism recently linked to CHAC1-mediated ferroptosis and apoptosis. The induction of NFE2L1 (NRF1) further implicates proteotoxic stress and compensatory lysosomal degradation responses, while the downregulation of TXNIP at 2.5 μM may reflect a transient adaptive attempt to buffer ROS accumulation that is overwhelmed at higher drug concentrations.^47^^,^^48^^,^^49^^,^^50^ The concentration-dependent induction of DDIT4 (REDD1) at 5 μM QNZ, an ATF4-driven physiological inhibitor of mTORC1, together with SESN2 upregulation at both doses, points to a convergent and dose-escalated suppression of mTORC1 signaling as a central transcriptional consequence of QNZ treatment. This is consistent with QNZ’s reported reduction of phosphorylated 4EBP1, p70S6K, and S6RP levels. The dose-dependent induction of DDIT4 may additionally reflect an adaptive resistance mechanism under conditions of maximal mTORC1 suppression, with implications for therapeutic dosing.^51^ The upregulation of ULBP1, a ligand for the activating NK cell receptor NKG2D, across all three MM cell lines suggests a potential immunostimulatory dimension to QNZ’s mechanism of action that may enhance NK cell-mediated cytotoxicity against drug-treated MM cells, warranting further investigation in immunocompetent co-culture models.^52^ Finally, a dose-dependent qualitative shift in enriched pathways is evident between 2.5 and 5 μM QNZ. The lower concentration favors broader stress-adaptive responses including redox homeostasis, cytokine signaling, and glucose metabolism, consistent with a partially adaptive transcriptional state. In contrast, the 5 μM signature is enriched for terminal processes such as L-serine and proteinogenic amino acid biosynthesis, cytoskeletal motor activity, and the CHOP-ATF3 complex, consistent with an apoptotic commitment and progressive proteostatic collapse. This dose-response dichotomy mirrors the dual role of UPR signaling in cancer, in which low-amplitude UPR activation is pro-survival while sustained high-amplitude UPR exceeds the apoptotic threshold and drives cell death.^53^ The transcriptomic analysis of primary MM patient samples revealed a patient-heterogeneous transcriptional response to QNZ, in line with the well-established molecular diversity of MM. Among the most prominently downregulated genes, SLC7A11 and GCLM are particularly noteworthy given their roles in antioxidant defense. SLC7A11 encodes the cystine/glutamate antiporter, whose NF-κB-dependent expression supports intracellular cystine import and glutathione biosynthesis, protecting cancer cells from oxidative stress and ferroptosis. GCLM, encoding the modifier subunit of glutamate-cysteine ligase, the rate-limiting enzyme in GSH synthesis, has similarly been linked to NF-κB-associated chemoresistance. Their co-downregulation upon QNZ treatment thus indicates a coordinated impairment of the GSH-mediated antioxidant axis, directly reflected in the GO enrichment of glutamate-cysteine ligase activity. The concurrent downregulation of SAP25 and ZNF75D further points to perturbation of chromatin-associated transcriptional repressor networks, consistent with the known capacity of NF-κB to remodel nucleosomal architecture at target gene loci.^54^^,^^55^^,^^56^ Taken together, these transcriptomic data delineate a mechanistically coherent, multi-layered program of QNZ-induced cytotoxicity in MM, centered on convergent activation of ER stress-driven apoptosis, metabolic reprogramming, mTOR suppression, oxidative stress amplification, and immune ligand upregulation, with a clear dose-dependent escalation toward terminal apoptotic commitment.

In previous studies, the NF-κB inhibitor QNZ has been shown to sensitize diverse tumor models to both targeted agents and conventional chemotherapies. QNZ targeted NFKB1 to overcome lenvatinib resistance in hepatocellular carcinoma and, in combination with cabozantinib (targeting MET), synergistically induced apoptosis and suppressed tumor growth *in vitro* and *in vivo*.^57^ Similarly, QNZ, or the related compound amentoflavone, potentiated sorafenib cytotoxicity in osteosarcoma by inhibiting NF-κB nuclear translocation and phosphorylation, thereby reducing metastatic traits, and it abrogated cisplatin-induced EGFR/NF-κB signaling to enhance cisplatin efficacy while diminishing invasion and promoting apoptosis in non-small cell lung cancer and oral squamous cell carcinoma.^58^^,^^59^^,^^60^ QNZ has also been reported to augment the microtubule inhibitor eribulin in MDA-MB-231 breast cells but, paradoxically, to increase NF-κB activity via TAB2 upregulation in breast MCF7 cells with consequent loss of sensitivity, underscoring lineage-specific effects.^61^ Additional work shows that QNZ sensitizes cells to glucose starvation through mTOR inhibition and cooperates with bevacizumab to suppress proliferation and mTOR activity in aggressive colon tumors, and that QNZ synergizes with the DNA-demethylating agent decitabine in classical Hodgkin lymphoma to enhance growth inhibition.^13^^,^^62^ Collectively, these findings support the concept that NF-κB blockade can broadly potentiate antitumor therapies across mechanistically distinct agents, while also highlighting the need for context-specific optimization and biomarker-guided scheduling.

Combination strategies are essential in MM management because they mitigate clonal heterogeneity and adaptive resistance, improve depth and durability of response, and permit therapeutic dose optimization to limit toxicity. Notably, several approved and investigational anti-myeloma agents converge on NF-κB signaling: BTZ stabilizes IκB proteins via proteasome inhibition, while IMiDs such as thalidomide and lenalidomide attenuate pro-inflammatory cytokine production (TNF-α, IL-1β, IL-6); additionally, compounds including MG132, curcumin, naringin, sorafenib, genistein, and parthenolide have been reported to suppress NF-κB in preclinical myeloma models, collectively underscoring NF-κB as a therapeutically tractable node in MM.^6^^,^^7^^,^^63^ Here, we provide the first evidence that QNZ potentiates the activity of established and next-generation anti-myeloma agents, supporting its further evaluation as a rational combination partner in anti-myeloma regimens. Our combination studies demonstrate that QNZ potentiates the activity of both proteasome inhibitors and IMiDs in MM cell lines, although the magnitude of synergy is clearly cell line- and time-dependent. BTZ showed pronounced synergism with QNZ in MM.1S cells and a progressive shift from antagonism to strong synergy in RPMI-S cells over 24–72 h (with the converse temporal pattern in JJN-3 cells), whereas CFZ produced largely consistent and robust synergistic cytotoxicity across cell lines and time points. Similarly, LEN and, more strikingly, POM displayed predominantly synergistic interactions with QNZ, with second-generation agents (CFZ, POM) yielding the most potent and reproducible effects. Among conventional agents, DOX converted from early antagonism to sustained synergy in MM.1S cells and was synergistic in the other lines, while DEX and MEL elicited mainly synergistic cytotoxicity (DEX showing greatest effect in MM.1S cells, MEL in RPMI-S cells). Collectively, these data support prioritizing QNZ combinations with second-generation proteasome inhibitors and IMiDs, as well as with DEX and MEL, for further mechanistic study and preclinical optimization, with careful attention to schedule- and genotype-specific effects.

In summary, the selective NF-κB inhibitor QNZ potently suppresses NF-κB-driven survival signaling in MM, reducing cell viability *in vitro*, in *ex vivo* samples, and in xenograft models, while inducing mitochondrial, caspase-dependent apoptosis and cell-cycle perturbation. Although bone marrow stromal contact partially attenuates its cytotoxicity, QNZ retains activity in co-culture models. Importantly, QNZ synergizes with novel anti-MM agents, including proteasome inhibitors and IMiDs (notably CFZ and POM), as well as with conventional agents such as DEX and MEL, highlighting its translational potential. These preclinical findings support further clinical evaluation of QNZ in combination regimens for MM.

## Materials and methods

### Reagents

The NF-κB inhibitor QNZ (EVP4593), (BTZ; Velcade), (CFZ; PR-171), (LEN; CC-5013), and (POM; CC-4043) were purchased from Selleck Chemicals (Houston, TX, USA). DOX, DEX, and MEL were obtained from Sigma-Aldrich (St. Louis, MO, USA).

### Culture of cell lines and primary cells

The MM cell line MM.1S was obtained from the American Type Culture Collection (ATCC, Manassas, VA, USA), and OPM-2 was acquired from the Deutsche Sammlung von Mikroorganismen und Zellkulturen (DSMZ, Braunschweig, Germany). The chemosensitive line RPMI-S and its drug-resistant sublines (RPMI-DOX6 and RPMI-DOX40 [DOX-resistant], RPMI-MR20 [mitoxantrone-resistant], and RPMI-LR5 [MEL-resistant]) were kindly provided by Dr. William S. Dalton (Moffitt Cancer Center, Tampa, FL, USA). Human MM cell lines OPM-1, KMS-11, and JJN-3 were generously donated by Dr. Teru Hideshima (Dana-Farber Cancer Institute, Boston, MA, USA). The human bone-marrow stromal cell line HS-5 was obtained from ATCC. All MM cell lines were maintained in RPMI-1640 medium (Cellgro/Mediatech) supplemented with 10% heat-inactivated fetal bovine serum (FBS; Harlan), 100 U/mL penicillin, 100 μg/mL streptomycin, and 2 mM L-glutamine (Gibco) at 37°C in a humidified atmosphere containing 5% CO2. HS-5 cells were cultured in Dulbecco’s modified Eagle’s medium (DMEM; Cellgro/Mediatech) supplemented with 10% heat-inactivated FBS, 100 U/mL penicillin, 100 μg/mL streptomycin, and 2 mM L-glutamine under the same incubation conditions.

Fresh MNCs were isolated from bone marrow aspirates of patients and from peripheral blood of healthy volunteers by Ficoll-Hypaque density centrifugation (Pharmacia). Patient MM cells were enriched by cell sorting using a CD138-PE monoclonal antibody to isolate CD138+ PCs and to separate non-plasma accessory cells from freshly obtained bone marrow samples. Primary cells were maintained in RPMI-1640 supplemented with 20% heat-inactivated FBS, 100 U/mL penicillin, 100 μg/mL streptomycin, and 2 mM L-glutamine at 37°C in 5% CO2. The study protocol (Myelom 001) was approved by the Biomedical Research Center Institutional Review Board, and informed written consent was obtained from all participants in accordance with the Declaration of Helsinki.

### Drug viability assays

The inhibitory effects of QNZ (EVP4593), administered alone or in combination with conventional or novel anti-MM (MM) agents, on MM cell viability were quantified using the MTT assay (Sigma-Aldrich, St. Louis, MO, USA). MM cell lines were seeded in 96-well plates at 1 × 10^4^ cells/well and exposed to a range of QNZ concentrations for 24, 48, and 72 h. Following treatment, formazan crystals were solubilized with 150 μL dimethyl sulfoxide (DMSO) and absorbance was measured at 540 nm with background reference at 690 nm on a microplate reader (Dynatech Lab Inc., Chantilly, VA, USA). Purified MM patient cells were plated in 384-well plates at 1 × 10^4^ cells/well and treated with serial concentrations of QNZ for 48 h at 37°C. Cell viability in these primary cells was assessed using the CellTiter-Glo (CTG) luminescent assay (Promega, Madison, WI, USA). After addition of the CTG reagent, the plates were incubated for 30 min and luminescence was recorded using a Luminoskan luminometer (Labsystems, Franklin, MA, USA). Peripheral blood mononuclear cells (PBMCs) were plated in 96-well plates at 1 × 10^5^ cells/well and treated with serial concentrations of QNZ for 48 h at 37°C. Cell viability was assessed by MTT assay as described earlier. Dose-response curves were analyzed to determine the concentration producing 50% inhibition of cell survival (EC50) using CalcuSyn software (Biosoft, Ferguson, MO, USA). Drug-drug interactions between QNZ and anti-MM agents were evaluated by isobologram analysis and calculation of the combination index (CI) according to the Chou-Talalay method using CalcuSyn: CI = 1 denotes additivity, CI < 1 indicates synergism, and CI > 1 indicates antagonism.

### Annexin V/PI-based apoptosis flow cytometry assay

Apoptotic cells were quantified using an Annexin V-FITC/PI assay. In brief, both suspension and adherent cells were harvested and washed twice with ice-cold phosphate-buffered saline (PBS). A total of 3 × 10ˆ5 cells were resuspended in 100 μL of the manufacturer-provided 1× binding buffer and incubated with 5 μL Annexin V-FITC (BD Biosciences Pharmingen, San Diego, CA, USA) and 5 μL PI. Following a 30-min incubation in the dark at room temperature (RT), samples were analyzed in a 96-well format on a fluorescence-activated cell sorting (FACS) Canto II flow cytometer (Becton Dickinson, Mountain View, CA, USA).

### JC-1-based mitochondrial membrane potential flow cytometry assay

The mitochondrial membrane potential of QNZ-treated and control MM cells was evaluated with the JC-1 fluorescent probe. JC-1 is a mitochondria-selective dye that forms aggregates in polarized mitochondria, producing orange fluorescence, whereas in cells with depolarized mitochondrial membranes JC-1 remains monomeric and emits green fluorescence. In brief, 3 × 10ˆ5 cells were incubated in 200 μL PBS supplemented with 0.2% bovine serum albumin (BSA) containing 4 μM JC-1 (Molecular Probes, Eugene, OR, USA) for 30 min at 37°C in the dark. After staining, samples were acquired in a 96-well format on a FACS Canto II flow cytometer (Becton Dickinson, Mountain View, CA, USA).

### PI-based cell cycle flow cytometry assay

Cell-cycle distribution in QNZ-treated cells was determined by flow cytometric quantification of cellular DNA content using PI staining. In brief, MM cells (3 × 10ˆ5) were harvested, washed twice with ice-cold PBS, and permeabilized by incubation with 0.05% Triton X-100 and 15 μL RNase A (10 mg/mL) for 20 min at 37°C. Samples were then cooled on ice for ≥10 min prior to the addition of PI (final concentration 50 μg/mL). DNA-stained cells were acquired in a 96-well format on a FACS Canto II flow cytometer (Becton Dickinson, Mountain View, CA, USA) for subsequent cell-cycle analysis.

### CFSE-based co-culture proliferation flow cytometry assay

To discriminate MM cells within co-culture systems, MM cells were labeled with (CFSE; Molecular Probes, Eugene, OR, USA), a cell-tracking dye that is partitioned equally between daughter cells upon division. Labeled MM cells were plated onto unlabeled bone marrow stromal HS-5 cells and cultured for 24, 48, and 72 h. Changes in mean fluorescence intensity (MFI) of the CFSE signal were used as a surrogate for cellular proliferation. In brief, MM cells were incubated with 1 μM CFSE in serum-free RPMI for 10 min at 37°C in the dark. The staining reaction was quenched by addition of RPMI-1640 supplemented with 2% FBS, and cells were washed three times with RPMI-1640 containing 10% FBS. Thereafter, CFSE-labeled MM cells were seeded either alone or onto unlabeled HS-5 stromal cells that had been plated 24 h earlier. Drug treatment experiments were subsequently performed using these co-culture systems.

### Flow cytometric acquisition and data analysis

Flow cytometry was performed on a FACS Canto II flow cytometer equipped with a 488 nm excitation laser. Fluorochromes were excited by the 488 nm laser and fluorescence was collected through the following photomultiplier tubes (PMTs): Annexin V-FITC and PI; CFSE, and 7-AAD (FL1, FL3); JC-1 (FL1 and FL2, with mitochondrial membrane potential reported as the FL2/FL1 ratio); and PI for cell-cycle analysis (log FL3 for sub-G1, linear FL2 for the DNA content histogram, and FL3 peak versus integral for doublet discrimination). Forward and side scatter parameters (FSC/saline sodium citrate, SSC) were used to exclude cellular debris. For each sample, 10,000–20,000 events were acquired. Data were processed and analyzed using De Novo FCS Express software (De Novo Software, Los Angeles, CA, USA).

### RNA-seq and transcriptome analysis

Total RNA was extracted from cell pellets using the RNeasy Mini Kit (QIAGEN, Hilden, Germany) according to the manufacturer’s instructions; with on-column DNase I digestion performed using the RNase-Free DNase Set (QIAGEN) to eliminate residual genomic DNA contamination. RNA integrity and purity were assessed spectrophotometrically using a NanoDrop ND-1000 spectrophotometer (Thermo Fisher Scientific, Waltham, MA, USA) by measuring absorbance ratios at 260/280 nm and 260/230 nm. RNA samples passing quality thresholds were subsequently subjected to paired-end sequencing (PE150) on the Illumina NovaSeq X Plus platform. RNA sequencing (RNA-seq) reads were aligned to the hg38 reference genome using the STAR aligner (Galaxy v.2.0.3) with default settings for paired-end reads.^64^ Gene-level counts were generated using the featureCounts tool (Galaxy v.2.0.3).^65^ Differential gene expression analysis was performed using the DESeq2 R package (v.1.50.2).^66^ *p* values were adjusted for multiple testing using the Benjamini-Hochberg method to control the FDR. Genes with an adjusted *p* value <0.05 were considered significantly differentially expressed. Subsequently, GO overrepresentation analysis was performed using the GO Enrichment tool (v.2.0.1) on the Galaxy web platform to identify enriched biological processes, molecular functions, and cellular components among the selected genes. The analysis was conducted using default settings, with an adjusted *p*-value threshold of 0.05. For the analysis of cell line samples, genes with |log2FC| > 1 and adjusted *p*-value <0.05 were included. For patient samples, due to the lack of statistically significant results, log2 fold changes were calculated for each sample individually between control and treated sample. Genes with log2FC > 1 and log2FC < −1 were then compared across samples to identify their intersections for each pair of samples as well as across all four patients. GO enrichment results were visualized using custom scripts in R (v.4.5.2) with the ggplot2 package.

### Western immunoblotting and detection of signaling protein

Following QNZ treatment, cells were washed twice with ice-cold PBS and lysed in 100 μL ice-cold cell lysis buffer (1% Nonidet P-40, 50 mM Tris, pH 7.4, 150 mM NaCl, 2 mM EDTA, 2 mM PMSF, 1 mM sodium vanadate, 1 mM sodium fluoride and a protease inhibitor cocktail). Lysates were incubated on ice for 20 min and clarified by centrifugation at 10,000× g for 10 min at 4°C. Supernatant protein concentrations were determined by Bradford assay. Equal amounts of protein (20 μg) were mixed with 4× SDS-PAGE sample buffer (Invitrogen, Carlsbad, CA, USA) and 10× reducing agent (0.5 M dithiothreitol; Invitrogen), resolved by SDS-PAGE and transferred to nitrocellulose membranes using a semi-dry transfer system. Membranes were blocked for 1 h at RT in 5% non-fat dry milk in Tris-buffered saline containing 1% Tween 20 (TBS-T, pH 7.4), then incubated overnight at 4°C with primary antibodies diluted 1:1,000 (Cell Signaling Technology, Danvers, MA, USA) against RelB, c-Rel, IκBα, phospho-IκBα (*p*-IκBα), IκBβ, IKK, NF-κB p105, NF-κB p65, phospho-NF-κB p65 (p-p65), NF-κB p50, mTOR, phospho-mTOR (*p*-mTOR), Akt, c-Myc, SIRT1, ATM, phospho-ATM (*p*-ATM), Chk2, phospho-Chk2 (*p*-Chk2), Cdc2, phospho-Cdc2 (*p*-Cdc2), phospho-4EBP1 (p-4EBP1), CDK4, cyclin A2, cyclin B1, Pro-caspase-9, Pro-caspase-8, Pro-caspase-3, Bcl-2, Mcl-1, Beclin-1, and GAPDH (loading control). After washing with TBS-T, membranes were incubated for 1 h at RT with horseradish peroxidase-conjugated goat anti-mouse or goat anti-rabbit secondary antibodies (1:10,000; Cell Signaling Technology). Immunoreactive bands were detected using an enhanced chemiluminescence (ECL) detection system (Amersham Bioscience, Little Chalfont, UK).

### Immunofluorescence analysis

Cells (controls and QNZ-treated) were deposited onto glass microscope slides by cytocentrifugation (cytospin). Slides were fixed in 4% paraformaldehyde in PBS for 20 min at RT, then washed three times with PBS. Cells were permeabilized with 0.1% Triton X-100 in PBS for 15 min at RT and washed a further three times with PBS. Non-specific binding sites were blocked by incubation in PBS containing 1% BSA and 5% normal goat serum for 1 h at RT. Slides were incubated overnight at 4°C in blocking buffer with the following primary antibodies: CD38-FITC, CD138-PE (clone MI15), cleaved caspase-3, and cleaved PARP (Cell Signaling Technology, Danvers, MA, USA). Unconjugated primary antibodies were detected by incubation with the appropriate fluorescently labeled secondary antibodies and phalloidin for 1 h at RT in the dark. Secondary-only controls were included to assess non-specific staining. Between antibody incubations, slides were washed four times with PBS. Nuclei were counterstained with DAPI (1 μg/mL; Molecular Probes) for 10 min at RT, followed by four PBS washes and a brief dip in deionized water to remove residual salts. A drop of anti-fade mounting medium was placed on a microscope slide and coverslips were mounted cell-side down. All procedures involving fluorophores were performed protected from light. Fluorescence images were acquired on an inverted Leica Mateo FL microscope (Leica Microsystems Inc., Buffalo Grove, IL, USA). Serial optical sections (50 μm) were collected and images were processed.

### s.c. xenograft implantation and treatment regimen

Mice were maintained in the Animal Research Facility of the Biomedical Research Center SAS, and all procedures were performed in accordance with approved institutional protocols. For the subcutaneous (s.c.) xenograft myeloma model, NSG mice were inoculated subcutaneously with RPMI-S cells (2.5 × 10ˆ6 cells in 100 μL PBS mixed with 100 μL Matrigel). After tumor engraftment, tumor-bearing animals were randomized into three groups (*n* = 6 per group). The control cohort received vehicle only (water containing 5% DMSO, 8% PEG300, and 5% Tween-80), whereas the treatment cohorts received QNZ at 1 or 2 mg/kg formulated in the same vehicle. Treatments were administered by oral gavage once daily for five consecutive days followed by two days off; this 7-day cycle was repeated for 4 weeks (5 on/2 off for 4 weeks). Animals were monitored every 2–3 days for changes in tumor burden, assessed by caliper measurement, and for body weight. Mice were euthanized per institutional humane endpoints when tumors reached 1.5 cm in diameter or if animals exhibited moribund appearance, to avoid unnecessary morbidity. Tumor volumes were calculated using the ellipsoid formula: V = (4/3)π × (a/2) × (b/2)ˆ2, where “a” and “b” represent the longest and shortest tumor diameters, respectively.

### Histology and IHC

Formalin-fixed, paraffin-embedded tumor samples were sectioned at 4 μm and stained with H&E for routine histology. For IHC, slides were deparaffinized, rehydrated, and subjected to antigen retrieval in high-pH Target Retrieval Solution (Dako) at 96°C for 20 min using a PT Link system. Endogenous peroxidase activity was blocked with FLEX Peroxidase Block (Dako) for 5 min. Slides were incubated at RT for 20 min with ready-to-use Ki-67 antibody (clone MIB-1, Dako), and overnight at 4°C with cleaved caspase-3 (Asp175) antibody (clone D3E9, Cell Signaling Technology) and CD138 (clone MI15, BioLegend) each diluted 1:250. Sections were then incubated with the EnVision+ System-HRP (Dako) for 15 min, visualized with 3,3′-diaminobenzidine (DAB) for 5 min, counterstained with hematoxylin for 10 min, and mounted with Faramount Aqueous Medium (Dako). Slides were examined using a Mateo FL microscope (Leica Microsystems, Germany).

### Apoptosis detection by TUNEL assay

TUNEL staining (In Situ Cell Death Detection Kit; Roche Diagnostics GmbH, Germany) was performed on formalin-fixed, paraffin-embedded (FFPE) mouse tumor sections to detect DNA fragmentation associated with apoptosis. Tissue sections (4–6 μm) were deparaffinized twice in xylene (10 min each), rehydrated through a graded ethanol series (96%, 70%, 50%; 3 min each) and rinsed in distilled water. Slides were permeabilized on ice for 2 min in 0.1% Triton X-100/0.1% sodium citrate, washed twice in PBS (3 min each), and briefly equilibrated in PBS. For each section, 50 μL of freshly prepared TUNEL reaction mix (prepared according to the manufacturer’s instructions) was added, and slides were incubated for 60 min at 37°C in a humidified, light-protected chamber, followed by three PBS washes. Sections were then mounted using a fluorescence mounting medium containing DAPI and examined using a fluorescence microscope (Mateo FL, Leica, Germany).

### Quantification of mouse plasma ALT by ELISA

Plasma was collected into heparinized tubes and centrifuged at 2,000× g for 10 min to remove cellular debris. Supernatants were diluted 1:20 in 1× Cell Extraction Buffer PTR. For the assay, 50 μL of each diluted sample, standard (46.88–1,500 pg/mL), or blank was added to the appropriate wells, followed by 50 μL of Antibody Cocktail. Plates were incubated for 1 h at RT on a plate shaker (400 rpm). Wells were then washed 3 times with 350 μL 1× Wash Buffer PT, after which 100 μL TMB Development Solution was added to each well and incubated in the dark for 10 min on a plate shaker (400 rpm). The reaction was stopped with 100 μL Stop Solution, the plate was mixed for 1 min on a shaker, and absorbance was read at 450 nm using a spectrophotometer (Dynatech Lab Inc., Chantilly, VA, USA).

### Statistical analysis

Statistical comparisons between QNZ-treated and control samples were performed by one-way analysis of variance (ANOVA) followed by Dunnett’s multiple-comparisons test using GraphPad Prism (GraphPad Software, La Jolla, CA, USA). Data are presented as mean ± standard deviation (SD). Statistical significance is denoted as ∗*p* < 0.05, ∗∗*p* < 0.01, ∗∗∗*p* < 0.001, and ∗∗∗∗*p* < 0.0001.

## Data and code availability

All data generated and analyzed in this study are included in this published article and its supplementary information.

## Acknowledgments

We thank Andrea Mlcakova from the National Cancer Institute and Tatiana Zeleznikova from St. Elizabeth Cancer Institute Hospital for providing research specimens. We also thank Peter Makovicky for assistance with histopathological analyses. This study was supported by the scientific grant agency VEGA 2/0088/23 (to J.J.), 10.13039/501100006109VEGA 2/0087/23 (to D.C.), 10.13039/501100006109VEGA 2/0127/26 (to J.J.); the 10.13039/501100005357Slovak Research and Development Agency APVV-20-0183 (to J.J.), APVV-21-0215 (to J.J.), APVV-24-0471 (to J.J.), APVV-19-0212 (to D.C.), APVV-23-0482 (to D.C.) grants, and the 10.13039/100031478NextGenerationEU project no. 09I03-03-V04-00451 (to J.J.) and no. 09I03-03-V02-00031 (D.C. and K.S.). This work was performed during the implementation of the building-up center for advanced materials application of the Slovak Academy of Sciences, ITMS project code 313021T081 supported by the Research & Innovation Operational Programme funded by the ERDF (to J.J.). This study was approved by the Institutional Human Ethics Committee of the Biomedical Research Center in Bratislava, Slovakia, under reference number Myelom 001. All patient and HD samples were obtained following written informed consent, consistent with the Declaration of Helsinki protocol. This study was approved by the Institutional Animal Ethics committee of animal research of the Biomedical Research Center in Bratislava, Slovakia, and State Veterinary and Food Administration of the Slovak Republic with reference number myelom. In accordance with institutional guidelines and approved protocols, *in vivo* experiments utilizing an MM xenograft mouse model were conducted.

## Author contributions

D.C. performed experiments, analyzed data, and contributed to writing of the manuscript; M.B. and E.S. performed experiments and analyzed data; M.H., G.B., and L.K. performed bioinformatic data analyses; Z.V., K.S., and G.G. performed experiments; J.S. contributed to the discussions; J.J. conceived and designed the study, performed experiments, analyzed data, and wrote the manuscript.

## Declaration of interests

The authors have no conflicts of interest to declare.

## Supplemental information

Document S1. Figures S1, S2, and Table S1

Table S2. QNZ-regulated transcriptome in multiple myeloma cell lines and primary patient samples Comprehensive lists of differentially expressed genes (DEGs) in MM.1S, RPMI-8226S, and JJN-3 MM cell lines treated with 2.5 or 5 μM QNZ compared to vehicle control. GO enrichment analysis of QNZ-regulated DEGs in all three cell lines at both treatment concentrations. DEGs and corresponding GO enrichment analysis identified in CD138⁺ PCs isolated from 4 primary MM patients treated *ex vivo* with 2.5 μM QNZ. Table S2 is provided as a separate excel file.

Document S2. Article plus supplemental information
