# Supplementary material for: Anti-myeloma mechanisms of the selective NF-κB inhibitor QNZ
Source: Mol Ther Oncol. 2026 Jun 12;34(3):201267. doi: 10.1016/j.omton.2026.201267 (PMC13333365; doi:10.1016/j.omton.2026.201267)
Supplement: Document S1. Figures S1, S2, and Table S1 [file mmc1.pdf]

OMTON, Volume *34*

Supplemental information

Anti-myeloma mechanisms of the selective NF-κB inhibitor QNZ

Dana Cholujova, Zuzana Valuskova, Monika Burikova, Milan Hucko, Gabor Beke, Eva Sedlackova, Lubos Klucar, Katarina Suroviakova, Gabriela Grofova, Jan Sedlak, and Jana Jakubikova

SUPPLEMENTAL INFORMATION

Table S1: **Clinical and laboratory characteristics of primary MM samples treated *ex vivo* with QNZ.** Clinical data were obtained from patients with a confirmed diagnosis of multiple myeloma and included demographic variables (age, sex), baseline biochemical parameters (serum M-protein, serum and urine free light chains, creatinine, calcium, hemoglobin, albumin, lactate dehydrogenase, and platelet count), prior treatment history (number of previous lines of therapy and type of prior treatments), and cytogenetic/molecular abnormalities.

CHARACTERISTICS	MM PATIENTS (N = 15)
Median age (range) - yrs	64 (45 - 78)
Sex - no. (%)	
Female	7 (47)
Male	8 (53)
Stage of myeloma - no. (%)	
NDMM	6 (40)
MM	9 (60)
Medium serum creatinine (range) - μmol/l	86.1 (58.6 - 340.6)
Medium calcium (range) - mmol/l	2.29 (2.1 - 2.97)
Medium serum albumin (range) - g/l	40.6 (24 - 49)
Medium lactate dehydrogenase (range) - μkat/l	2.7 (1.02 - 7.02)
Hemoglobin (range) - g/l	139 (91 - 152)
Platelet count (range) - G/l	191.5 (102 - 358)
Type of myeloma (immunoglobulin)	
Median IgA (range) - mg/dl	1.5 (0.3 - 13.2)
no. of patients (%)	3 (20)
Median IgG (range) - mg/dl	10.6 (4.4 - 87.8)
no. of patients (%)	12 (80)
Type of light chain	
Median kappa (range) - g/l	3.7 (1.1 - 10.4)
no. of patients (%)	9 (60)
Median lambda (range) - g/l	1.2 (0.18 - 4.63)
no. of patients (%)	6 (40)
Median k/l (range)	4.18 (0.27 - 41.61)
Median bone marrow involvement (range) - (%)	30 (10 - 50)
Previous therapy	
Median no. of previous treatment regimens (range)	2 (0 - 6)
Type of previous therapy - no. (% of treated MM)	
CVD (Cyclophosphamide-Velcade-Dexamethasone)	3 (33)
RCd (Revlimid-Cyclophosphamide-Dexamethasone)	4 (44)

HD CHT+ASCT/ASCT	5 (56)
R/Rd (Revlimid/Revlimid-Dexamethasone)	2 (22)
Pd (Pomalidomide- Dexamethasone)	1 (11)
VMP senior (Velcade-Melphalan-Prednisone)	1 (11)
DVd (Daratumumab-Velcade-Dexamethasone)	1 (11)
VAD (Vincristine-Doxorubicin-Dexamethasone)	1 (11)
Cytogenetic abnormalities - no. (%)	
t(4;14)	1 (7)
del(13)	1 (7)
del(17)	2 (13)
del(17p)	2 (13)
CyclinD1+	5 (33)
Hyperdiploidy	6 (40)

Table S2: **QNZ-regulated transcriptome in multiple myeloma cell lines and primary patient samples.** Comprehensive lists of differentially expressed genes (DEGs) in MM.1S, RPMI-8226S, and JJN-3 MM cell lines treated with 2.5 μM or 5 μM QNZ compared to vehicle control. Gene Ontology (GO) enrichment analysis of QNZ-regulated DEGs in all three cell lines at both treatment concentrations. DEGs and corresponding GO enrichment analysis identified in $CD138^{+}$ plasma cells isolated from 4 primary MM patients treated ex vivo with 2.5 μM QNZ. Table S2 is provided as a separate excel file.

A

Cell cycle pathway

	MM.1S					RPMI-S					JJN-3				
	0	2.5	5	10	20	0	2.5	5	10	20	0	2.5	5	10	20
SIRT1	1.00	1.00	0.84	0.79	0.87	1.00	0.99	0.84	0.76	0.65	1.00	0.92	0.67	0.60	0.61
ATM	1.00	1.20	1.00	0.96	1.23	1.00	1.12	1.10	1.04	0.95	1.00	1.00	0.76	0.62	0.65
p-ATM	1.00	1.54	1.41	1.14	0.97	1.00	1.55	1.62	1.65	1.64	1.00	0.85	0.80	0.74	0.59
Chk2	1.00	0.16	0.14	0.12	0.11	1.00	1.54	0.59	0.06	0.05	1.00	0.05	0.17	0.03	0.03
p-Chk2	1.00	0.19	0.07	0.07	0.05	1.00	0.53	0.42	0.40	0.53	1.00	0.41	0.14	0.10	0.11
Cdc2	1.00	0.81	0.81	0.81	0.75	1.00	0.88	0.84	0.82	0.64	1.00	1.24	0.95	0.65	0.69
p-Cdc2	1.00	1.05	1.04	1.01	1.01	1.00	1.04	0.96	0.84	0.76	1.00	1.10	0.94	0.98	0.94
p-4EBP1	1.00	1.26	1.31	0.85	0.11	1.00	1.05	0.44	0.19	0.16	1.00	0.37	0.14	0.02	0.01
CDK4	1.00	1.15	0.96	1.04	0.98	1.00	1.00	0.85	0.75	0.72	1.00	0.93	0.73	0.71	0.76
Cyclin A2	1.00	0.90	0.53	0.46	0.58	1.00	1.17	1.04	1.08	0.88	1.00	0.73	0.69	0.65	0.67
Cyclin B1	1.00	0.89	0.74	0.74	0.62	1.00	1.05	1.01	0.86	0.77	1.00	0.87	0.81	0.65	0.91
GAPDH	1.00	0.99	1.07	1.14	1.04	1.00	1.05	1.02	0.95	0.94	1.00	0.95	0.92	0.86	0.87

>=2; 1.76 - 1.99; 1.51 - 1.75; 1.25 - 1.50; 0.76 - 1.24; 0.51 - 0.75; 0.26 - 0.50; <=0.25

B

Apoptotic pathway

	MM.1S					RPMI-S					JJN-3				
	0	2.5	5	10	20	0	2.5	5	10	20	0	2.5	5	10	20
Pro-Caspase-9	1.00	0.23	0.32	0.71	0.51	1.00	0.74	1.06	0.02	0.09	1.00	0.56	0.18	0.02	0.01
Pro-Caspase-8	1.00	0.78	0.54	0.56	0.52	1.00	1.01	0.71	0.49	0.57	1.00	0.43	0.26	0.13	0.02
Pro-Caspase-3	1.00	0.90	0.77	0.51	0.54	1.00	1.07	0.46	0.70	0.64	1.00	0.88	0.79	0.56	0.66
cl. Caspase-3	1.00	115.03	269.74	89.46	12.08	1.00	1.00	1.00	1.00	1.00	1.00	36.90	152.59	120.96	75.29
Bcl-2	1.00	1.03	0.97	0.96	0.91	1.00	0.88	0.87	0.96	0.96	1.00	0.91	0.86	0.92	0.64
Mcl-1	1.00	1.08	0.94	0.98	1.00	1.00	1.00	0.88	0.73	0.67	1.00	0.72	0.55	0.55	0.61
Beclin-1	1.00	1.12	0.94	0.87	0.85	1.00	1.02	0.97	0.83	0.81	1.00	0.94	0.99	0.99	0.92
GAPDH	1.00	1.04	1.09	1.10	1.24	1.00	1.01	1.01	1.01	0.99	1.00	1.09	0.96	1.04	1.15

>=2; 1.76 - 1.99; 1.51 - 1.75; 1.25 - 1.50; 0.76 - 1.24; 0.51 - 0.75; 0.26 - 0.50; <=0.25

C

NF-κB pathway

	MM.1S					RPMI-S					JJN-3				
	0	2.5	5	10	20	0	2.5	5	10	20	0	2.5	5	10	20
mTOR	1.00	0.39	1.76	1.49	1.71	1.00	1.51	1.54	1.29	1.32	1.00	1.06	1.12	1.33	1.38
p-mTOR	1.00	0.99	1.71	1.38	1.61	1.00	3.06	3.48	3.34	3.00	1.00	1.37	1.70	1.25	1.06
Akt	1.00	1.07	1.04	1.07	1.07	1.00	0.88	0.74	0.69	0.65	1.00	0.82	0.86	0.83	0.70
c-Rel	1.00	0.90	0.67	0.53	0.68	1.00	0.89	0.79	0.73	0.75	1.00	0.85	0.88	0.95	0.81
RelB	1.00	1.05	0.78	0.66	0.56	1.00	0.94	0.78	0.64	0.73	1.00	0.79	0.60	0.52	0.33
NFkBp105	1.00	1.21	0.94	1.00	0.46	1.00	1.68	1.67	0.57	0.57	1.00	0.90	0.49	0.44	0.28
NFkBp65	1.00	0.82	0.62	0.61	0.62	1.00	0.95	0.79	0.70	0.60	1.00	0.89	0.72	0.73	0.55
NFkBp50	1.00	1.09	1.11	1.08	1.13	1.00	1.07	1.10	1.00	0.98	1.00	1.06	0.93	0.86	0.77
IkBa	1.00	0.63	0.48	0.39	0.33	1.00	0.95	0.75	0.57	0.46	1.00	0.68	0.42	0.38	0.07
IkBb	1.00	1.04	0.59	0.49	0.56	1.00	0.84	0.67	0.38	0.81	1.00	0.46	0.21	0.25	0.31
IKK	1.00	0.85	0.84	0.83	0.78	1.00	1.02	0.89	0.86	0.93	1.00	1.10	1.09	0.98	1.19
c-Myc	1.00	0.80	0.56	0.62	0.63	1.00	0.84	0.67	0.27	0.22	1.00	0.70	0.36	0.15	0.17
GAPDH	1.00	1.05	1.04	1.01	0.97	1.00	0.96	0.97	0.97	1.02	1.00	0.98	0.99	0.93	0.96

>=2; 1.76 - 1.99; 1.51 - 1.75; 1.25 - 1.50; 0.76 - 1.24; 0.51 - 0.75; 0.26 - 0.50; <=0.25

Figure S1: **Quantification of western blot signals.** Densitometric quantification of the western blot data presented in (A) Figure 2B, (B) Figure 2C and (C) Figure 3A was carried out using ImageJ software to assess protein expression levels.

A

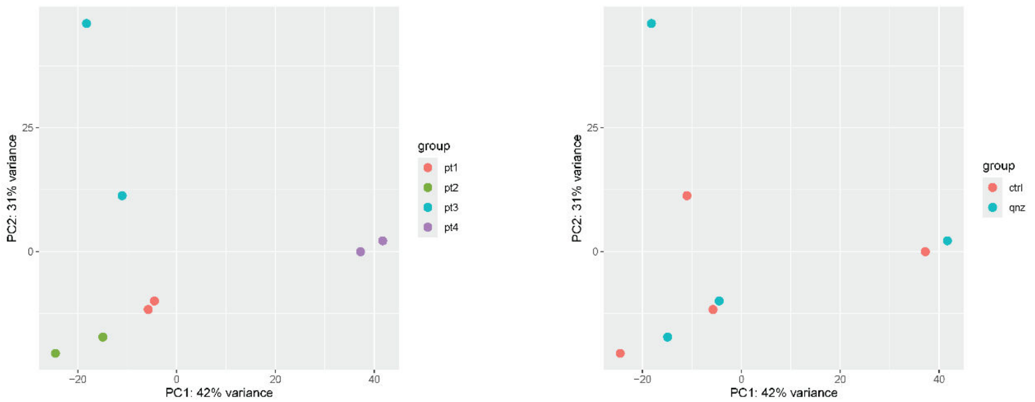

B

● pt1 ● pt2 ● pt3 ● pt4

baseMean	log2FoldChange	lfcSE	stat	p value	p adj	gene
●●●●						
65.77	-9.27	2.00	-4.64	3.5219E-06	0.0387	SAP25
●●●						
73.45	-9.39	1.80	-5.22	1.8271E-07	0.0023	ZNF75D
533.20	-3.59	0.77	-4.67	3.0458E-06	0.0189	GCLM
55.63	-8.99	2.01	-4.48	7.3857E-06	0.0305	AMN1
1124.70	-3.27	0.74	-4.42	9.9814E-06	0.0310	SLC7A11
39.41	9.11	2.09	4.36	1.3182E-05	0.0327	MPIG6B
195.91	5.85	1.36	4.31	1.6045E-05	0.0332	PRG3
45.70	-8.71	2.05	-4.25	2.1136E-05	0.0375	HPS3

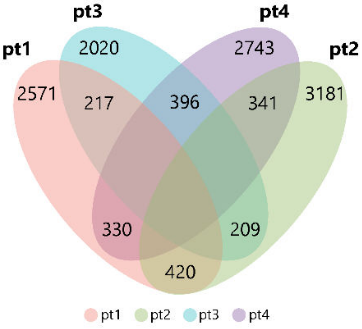

C

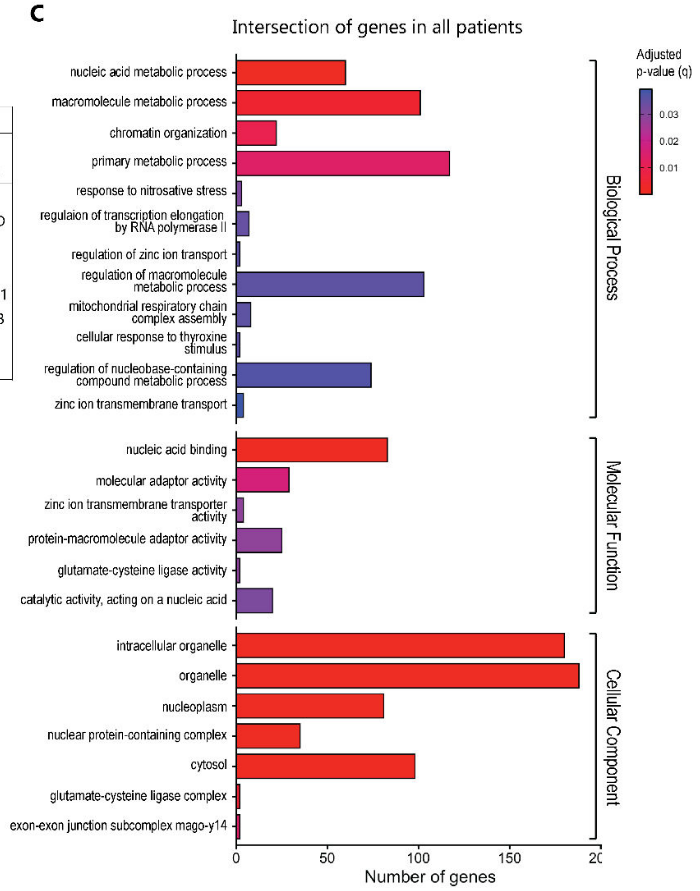

Figure S2. **Transcriptomic profiling of QNZ-treated primary MM patient samples.** (A)

Principal component analysis (PCA) of RNA-seq data from four MM patient samples (pt1 - pt4) treated with 2.5 μM QNZ or vehicle control. Left panel: samples colored by patient identity; right panel: samples colored by treatment group (ctrl, control; qnz, QNZ-treated). PC1 and PC2 explain 42% and 31% of the total variance, respectively. (B) Table of the significant top differentially expressed genes across patient samples (pt1 - pt4 or pt1,2,4), showing baseMean, $\log_2$ fold change ($\log_2$FC), lfcSE, Wald statistic, p-value, adjusted p-value (p adj), and gene symbol. Venn diagram depicting the overlap of differentially expressed genes among four MM patient samples (pt1 - pt4), with numbers indicating genes shared between each pairwise combination of patients, as well as genes uniquely deregulated in each individual patient. (C) Gene Ontology (GO) enrichment analysis of commonly deregulated genes, categorized by Biological Process, Molecular Function, and Cellular Component. Bar length indicates the number of genes; color scale represents the adjusted p-value (q).
